# Dissecting the sequence and structural determinants guiding m6A deposition and evolution via inter- and intra-species hybrids

**DOI:** 10.1186/s13059-024-03182-1

**Published:** 2024-02-15

**Authors:** Ran Shachar, David Dierks, Miguel Angel Garcia-Campos, Anna Uzonyi, Ursula Toth, Walter Rossmanith, Schraga Schwartz

**Affiliations:** 1 Department of Molecular Genetics, Weizmann Institute of Science, Rehovot, 7630031 Israel; 2 Center for Anatomy & Cell Biology, Medical University of Vienna, Vienna, 1090 Austria

## Abstract

**Background:**

N6-methyladenosine (m6A) is the most abundant mRNA modification, and controls mRNA stability. m6A distribution varies considerably between and within species. Yet, it is unclear to what extent this variability is driven by changes in genetic sequences (‘cis’) or cellular environments (‘trans’) and via which mechanisms.

**Results:**

Here we dissect the determinants governing RNA methylation via interspecies and intraspecies hybrids in yeast and mammalian systems, coupled with massively parallel reporter assays and m6A-QTL reanalysis. We find that m6A evolution and variability is driven primarily in ‘cis’, via two mechanisms: (1) variations altering m6A consensus motifs, and (2) variation impacting mRNA secondary structure. We establish that mutations impacting RNA structure - even when distant from an m6A consensus motif - causally dictate methylation propensity. Finally, we demonstrate that allele-specific differences in m6A levels lead to allele-specific changes in gene expression.

**Conclusions:**

Our findings define the determinants governing m6A evolution and diversity and characterize the consequences thereof on gene expression regulation.

**Supplementary Information:**

The online version contains supplementary material available at 10.1186/s13059-024-03182-1.

## Background

M6A is a highly abundant modification on mRNA, occurring at roughly ~ 0.2–0.4% of all adenosines [1], likely at hundreds of thousands of residues transcriptome-wide. Its deposition is strongly and causally associated with mRNA destabilization [2–6], and hence understanding the determinants governing its deposition (the ‘m6A code’) and evolution are of intense interest in the quest for understanding the forces shaping mRNA levels within cells- in health and disease. The m6A code is understood to a limited extent. A key component required for methylation is a methylation consensus motif, whose core is often represented as a DR**A**CH motif in mammalian cells (D = A/G/U, R = A/G, H = A/C/U) or DRAC in yeast cells, and which also extends into adjacent nucleotides, with a preference for an A and a U at positions -4 and + 4, respectively [7, 8]. In previous work we estimated that roughly 33%-46% of the variability in methylation levels between sites can be predicted on the nucleotide composition in these surrounding positions. While this suggested that a m6A deposition is to a substantial extent ‘hard coded’ in cis, it left open the question as to additional determinants guiding deposition of m6A and underlying the remaining variability. An additional recent advance has been the understanding that in mammals, in order to undergo methylation, a DRACH motif must reside at a distance of > 100 nt from a splice junction [6, 9, 10].

An additional feature, thought to impact m6A but potentially in a complex manner, is mRNA secondary structure. RNA secondary structure was found to inhibit m6A formation under in-vitro settings on the basis of purified METTL3-METTL14 heterodimers [11]. Yet, predicted mRNA secondary structures were shown to correlate negatively with m6A levels measured in human cells [7, 8], but to correlate positively in another [12]. In both cases the correlations were weak in nature, raising questions as to the in-vivo relevance of secondary structure on m6A formation, in particular given that it has been a long-standing question as to the extent to which secondary structures actually occur in-vivo, among others given ATP-dependent processes unfolding RNA [13–16]. Notably, interpretation of associations between mRNA secondary structure and methylation are rendered challenging, as in addition to the impact of structure on m6A, m6A can also impact RNA structure [16–19].

Our limited understanding of the m6A code has constrained our understanding of how m6A evolves, both between different species and between different individuals of the same species. Substantial differences are present between the human and mouse methylomes [20], as in the methylomes of the two yeast species *Saccharomyces cerevisiae* and *Saccharomyces mikatae *[8]. Yet, the factors driving these evolutionary changes are poorly understood. Two studies have also generated m6A maps across dozens of human individuals [21, 22], finding hundreds to thousands of sites with varying m6A levels between individuals which were significantly associated with genetic polymorphisms (‘m6A-QTLs’). Interestingly, only ~ 18% of the significant m6A-QTLs disrupted or created a DRACH motif [22], whereas the mechanism connecting the vast majority of polymorphism with m6A levels remained unaccounted for. Thus, we lack a full mechanistic understanding connecting sequence variation with m6A evolution.

**Fig. 1:**
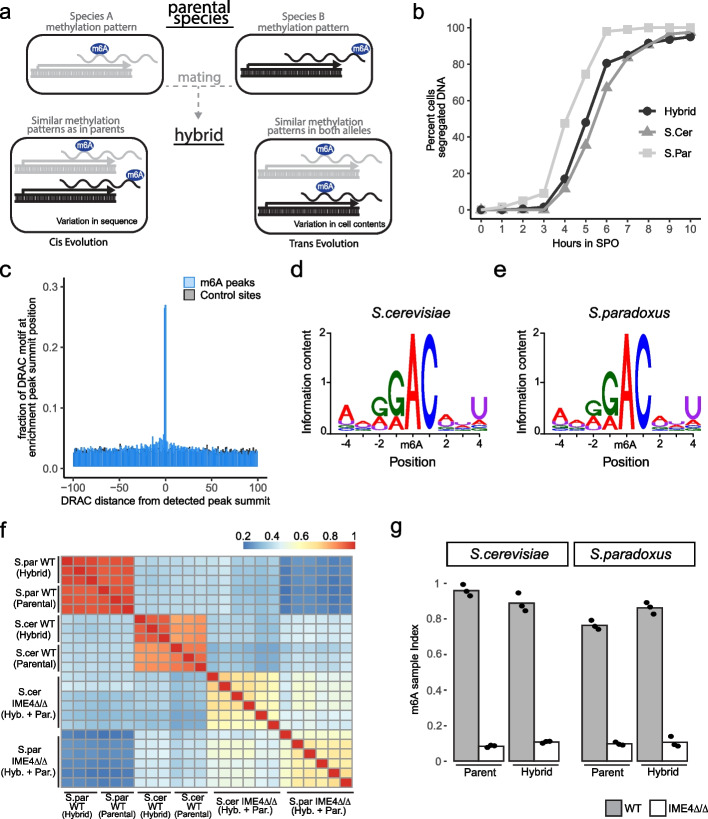
Establishment of *S. cerevisiae*—*S. paradoxus* hybrid as a model to study m6A regulation. **a** schematic representation of hybrids as a system to dissect cis and trans evolution of m6A regulation. Any differences in m6A levels across the two alleles in the hybrid are defined as changes in cis. Differences between the methylation patterns across the parental strains that are not maintained across the two alleles in the hybrid are considered trans effects. **b** Kinetics of meiotic progression across the parental and hybrid strains used in this study. DNA DAPI staining depicts the nuclear division at different time points (x-axis) after incubation of cells in a sporulation medium (SPO). n = 200. **c** The relative frequency of DRAC motif along a 200 nt window centered around the m6A enrichment peak summit positions (blue), in comparison to randomly sampled regions across the same genes (gray). The median distance of the identified sites from a consensus site is 1 nt, compared to 14 nt for randomly sampled ones.** d-e** Sequence logo of the methylation consensus sequence based on 975 m6A sites detected in S.cer alleles (**e**) or 767 m6A sites identified in S.par alleles. We filtered for m6A peak summits that were within 2 nt from DRAC site. **f** Clustered pairwise correlation matrix of m6A peak intensities across the different backgrounds. **g** m6A sample index scores for the parental strains and the two alleles of the hybrid strain in the WT and ime4-deletion strains. The m6A enrichment score is calculated as the normalized sum enrichment of m6A-IP versus input reads across all of the detected m6A sites identified in any of the backgrounds. Each background was measured in three biological replicates, each biological replicate is displayed as a point

In principle, evolution between or within species can be directed either via mutations in local regulatory sequences (‘cis’), or via regulators (‘trans’), or via a combination of both [23, 24]. In the context of m6A, an example of a ‘cis’ change could be a DNA mutation eliminating the DRACH motif from the transcribed mRNA, whereas an example of a ‘trans’ change would be a modulation in the specificity of an m6A writer (or eraser). Interspecies hybrids, harboring two distinct parental alleles within a single ‘trans’ environment, offer an attractive model to systematically distinguish ‘cis’ effects from ‘trans’ formally. Any difference between two parental alleles in the (shared) hybrid environment is considered changes ‘in cis’. In contrast, changes between the two parental alleles that are not maintained in the hybrid are attributable to differences in the ‘trans’ environment (Fig. 1a). Thus, dissection of m6A deposition via interspecies hybrids allows formally inferring whether sites are regulated ‘in cis’ or ‘in trans’, without making any assumptions about the presence of predefined sequence motifs (indeed: such motifs can be at considerable distance from the modified site), and as such offers a powerful lens via which to dissect the m6A code. This notwithstanding, an additional strength of interspecies hybrids is that the two similar yet distinct alleles can be conceptualized as a large-scale mutagenesis experiment. In vivo mutagenesis of individual m6A sites is tedious and challenging to perform at a systematic scale. The hybrid genome contains thousands of sites with minor differences in the sequence between the alleles, and careful comparison of signals originating from such related alleles may allow unraveling the determinants underlying the differences between the two. Thus, interspecies hybrids offer a naturally attractive model for studying the global determinants of the m6A code and of its evolution.

Here we employ both intra- and inter-species hybrids in yeast and mammalian systems to explore the evolution of m6A, and unravel the determinants impacting its deposition. Via careful mapping of m6A at near single-nucleotide resolution, we demonstrate that changes in m6A deposition between closely related yeast or mammalian species are frequent, occurring at thousands of sites transcriptome-wide. Interestingly, we demonstrate that these changes are nearly entirely (95% in yeast, 71% in mammals) driven ‘in cis’, with the majority of changes (68% in yeast, 59% in mammals) occurring due to disruption of the methylation consensus motif in one of the alleles. Yet, in > 30% of the cases in both yeast and mammals, dramatic changes are observed between the two alleles, despite an intact methylation consensus motif. We uncover that the majority of these changes are due to changes in local secondary structure, whereby a relaxation of secondary structure is associated with the accumulation of m6A and vice versa. We demonstrate that secondary structure is causal, and that gain of structure can be sufficient to abrogate methylation, whereas loss of secondary structure can be sufficient to gain m6A, thereby establishing a mechanism for how mutations occurring at regions distal to the methylation site can impact m6A formation. Finally, we find that the rules guiding m6A evolution between species also underlie differences in m6A distribution within a species, and provide a mechanistic basis for many previously observed m6A-QTLs associated with differences in m6A levels between humans. Collectively, our findings provide a broad overview of the cis-mediated evolution of m6A within and between species.

## Results

### Yeast hybrid as a model organism to study m6A RNA modification regulation

To unravel the determinants underlying m6A evolution, we first sought to monitor its evolution among two related yeast species, *Saccharomyces cerevisiae* and *Saccharomyces paradoxus*, on the basis of an interspecies hybrid. These two yeast species diverged roughly 5 million years ago and share 80% and 90% sequence similarity in the coding and intergenic regions, respectively [25]. This genetic divergence is distant enough to have allowed for many substitution to accumulate, yet close enough to render a comparison informative, and interspecies hybrids of these two species have therefore been leveraged in the past to dissect ‘cis’ and ‘trans’ determinants governing, among others, gene expression, translation efficiency, nucleosome positioning [23, 26, 27]. Nonetheless, a unique challenge for dissecting the determinants of mRNA methylation was that in budding yeast, mRNA methylation occurs at appreciable levels only during meiosis. Meiosis is triggered, at varying efficiencies, in diploid yeast following starvation for nitrogen and fermentable carbon sources and is coupled to sporulation. To study m6A, it was thus critical to establish an interspecies hybrid capable of efficiently and synchronously undergoing meiosis. We tested four hybrids of different *S. cerevisiae* and *S. paradoxus* parental strains, and by tracking DNA content after meiosis induction, we were able to identify one hybrid strain derived from the parental strains SK1 (*S. cerevisiae*) and YPS138 (*S. paradoxus*) that underwent rapid sporulation at nearly 100% efficiency (Fig. 1b and Additional file 1: Fig. S1a,b). For each of the two parental strains and for the hybrid, we next generated two mutants: (1) ndt80Δ/Δ strains, deleted of the critical transcription factor ndt80, which is required for entry into the meiotic divisions; In its absence, cells are synchronized at meiotic prophase, during which m6A levels are maximal [8, 28]. We will refer to this set of strains as ‘WT’. (2) ndt80Δ/Δ and ime4Δ/Δ strains, also deleted of ime4, the catalytic component of the methyltransferase complex in yeast, which is required for methylation [8, 28]. We will refer to this set of strains as ‘Δime4’.

### Optimization of m6A-seq2 to yield nearly single-nucleotide resolution data

m6A-seq2 [5], a protocol allowing multiplexed IP-based measurement of m6A across a pre-barcoded pool of samples, offers important advantages in terms of scalability, elimination of batch effects, and quantification of m6A levels at varying resolutions (site, gene, sample), all of which rendered it an attractive method of choice for obtaining m6A readouts. Nonetheless, a key limitation of this methodology is its resolution, given that it relies on antibody-based enrichment of methylated fragments. Given that a key motivation of this study was to investigate the determinants giving rise to variability in m6A levels at individual sites, we invested considerably in optimizing the resolution of the m6A-seq2. We found that optimizations on both the experimental and on the analytic end could, in combination, substantially enhance the resolution of the output, rendering it nearly single-nucleotide resolution. Specifically, two key improvements of the protocol included: (1) a sequential, two step m6A-IP, using two different m6A antibodies, and (2) stringent computational filtering of the data. As demonstrated below, ~ 57% of the peaks called by this optimized approach in both yeast and mammalian samples were within a single nucleotide (at most) of an m6A motif (DRAC and DRACH in yeast and mammals, respectively), and 77% were within 5 nt of the m6A motif. Thus, the resolution of the readouts by this enhanced protocol are close to ones achieved by m6A-eCLIP approach, where ~ 62% of detected sites in human were within one nt of a DRACH motif [29]. Consistently, roughly 75% of the sites detected in human cells were also identified on the basis of either published m6A-miCLIP datasets or the more recently published GLORI datasets (or both), attesting to the specificity and resolution of the mapping using this optimized approach [30–32].

### Application of optimized m6A-seq2 to yeast samples

We next induced synchronous meiosis across three biological replicates across each of the four parental strains (WT & Δime4 for each of *S. cerevisiae* and *S. paradoxus*) and the two interspecies hybrid strains (WT & Δime4). Prophase-synchronized cells were harvested six hours after meiosis induction, and subjected to optimized m6A-seq2. IP and Input libraries were sequenced to a depth of ~ 10 million reads/library and aligned to a single assembly comprising the full *S. cerevisiae* and *S. paradoxus* genomes. The vast majority of the aligned reads (95%) mapped uniquely to a single locus, and of these the overwhelming majority (~ 98%) were assigned to the proper reference allele, as was judged based on the alignment of reads originating from the parental species. Reads from the hybrid sample aligned equally between the chromosomes, as expected (Additional file 1: Fig. S1c).

We applied a peak-calling approach to data from both parents and the hybrid. As indicated above, we invested considerably in peak-calling, building on our previously described strategy [7, 8], but with stringent criteria for local enrichment detection and reproducibility across replicates, and included filtering of all sites detected in the IME4-KO strain (Methods). We identified a total of 2482 ‘m6A peaks’, which were highly enriched towards gene ends, consistent with previous reports ([7, 8] and Additional file 1: Fig. S1d). The resultant catalog allowed near single-nucleotide resolution inference of precise methylated positions, with 26.7% of the peaks were centered precisely over a DRAC motif (the random expectation for this is 2.4%), and 57% of the sites being within a single nucleotide from adenosine within a DRAC motif (Fig. 1c).

Based on these results, each ‘peak’ was assigned to the nearest DRAC consensus motif, which was considered a putative methylated site. The m6A consensus motifs in *S. cerevisiae* and *S. paradoxus* were essentially indistinguishable (Fig. 1d-e), and similar to the previously reported m6A consensus sequence in yeasts [7, 8]. Peak intensities correlated very well with each other in replicate experiments and also exhibited excellent correlations between the identical alleles in the parental and hybrid strains (Fig. 1f). Peak intensities were substantially less correlated between the two different alleles (Fig. 1f), hinting at considerable changes in methylation landscapes between the two. A global assessment of m6A levels, on the basis of the ‘m6A sample index’ [5] revealed that the interspecies hybrid is methylated to similar levels as the parental species (Fig. 1g).

### Cis-regulatory elements guide the evolution of m6A in yeast species

**Fig. 2:**
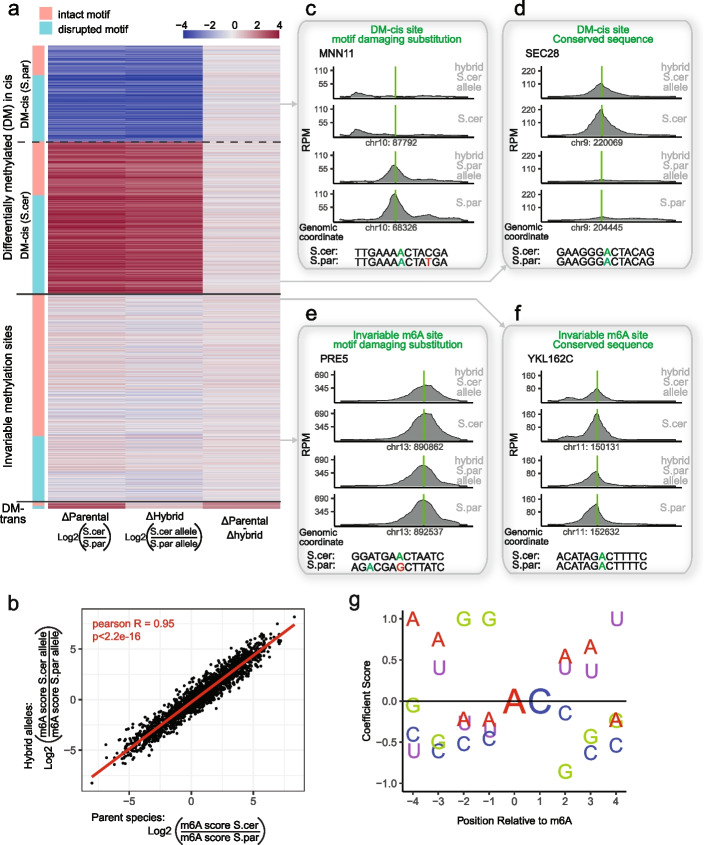
The evolution of m6A sites in yeast is determined mainly by cis-elements. **a** Classification of the detected m6A sites in yeast into significant differentially methylated sites in cis (DM-cis) or trans (DM-trans) (absolute log2 fold change > 1.6) or into ‘invariable’ m6A sites. The left column depicts the difference in m6A levels between the two parental strains. A site labeled in red is methylated at higher levels in S. cerevisiae than in *S. paradoxus*, a site labeled in blue is methylated higher in *S. paradoxus*, whereas sites in various shades of white show little difference between the alleles. The middle column depicts the corresponding difference between the two alleles of the hybrid. The right column displays the difference between the first two columns. The color bar to the left of the heatmap indicates whether a mutation disrupting the sequence motif evolved between the two species (peach), or not (turquoise). Δparental corresponds to [log2(m6A site score S.cer)-log2(m6A site score S.par)], while ΔHybrid calculated as [log2(m6A site score S.cer allele)-log2(m6A site score S.par)]. **b** Differences in m6A levels across the two hybrid alleles (Y-axis) as a function of difference at corresponding sites in the parental strains (X-axis) across the 2349 identified m6A sites. **c-f** Sequence coverage plots of m6A-seq2 IP for S.cer, S.par, and their corresponding alleles in the hybrid. Examples of the four main types of m6A sites identified in this study are shown: DM-cis sites in which the m6A consensus motif evolved a mutation (**c**), DM-cis sites in which the consensus motif did not evolve a mutation (**c**), Invariable sites in which the m6A consensus motif did not evolve a mutation (**e**) and invariable sites in which the m6A consensus motif did evolve a mutation (**f**). Green bars indicate the position of the detected m6A and its homologous locus on the other species. The 13-nt sequence in each of the two strains/alleles is shown on the bottom, indicating the methylated adenosine (green), a motif-damaging mutation (red), or evolved m6A motif on the other species (green). **g** Assessment of relative essentiality of sequence composition for methylation. For each differentially methylated site harboring a mismatch between *S. paradoxus* and *S. cerevisiae*, we calculated the frequency of each mutation from any base into any other base for each of the nucleotides centered around the methylation motif, reasoning that such mutations are particularly disruptive. These frequencies were normalized by their counterparts in the m6A conserved set of sites, with the rationale that these should reflect ‘neutral’ changes with respect to m6A formation. The coefficient score is calculated as the reduction of the per-base nucleotide conservation rate in ‘differentially -methylated cis’ versus ‘invariable’ m6A sites, normalized around 0 and scaled between -1 to + 1

We next compared the observed methylation maps between the two parental species (Fig. 2a, left column). We found that differences in methylation status between the two parental alleles were common. ~ 45% of the detected sites were conserved between the two (‘Invariable methylation’), whereas ~ 55% were differentially methylated (‘Differentially Methylated, DM). Of note, given the strict criteria that we employed for defining a site as differentially methylated (> threefold difference in enrichment levels), this likely underestimates the number of differentially detected sites. Thus, despite the global similarity in methylation levels (Fig. 1g), the individually methylated sites evolved considerably between the species.

Remarkably, the differences in methylation levels between the parental strains (Fig. 2a, left column) were nearly indistinguishable from the differences between the two alleles in the hybrid strain (Fig. 2a, center column). Consistently, the difference in these differences was by and large close to 0 (Fig. 2a, third column). Indeed, a correlation of changes in methylation between the hybrid alleles against changes in the respective parental strains yielded an *R* = 0.95 (Fig. 2b), indicating that ~ 91% (0.95^2^) of the differences in m6A patterns between *S. cerevisiae* and *S. paradoxus* are due to changes ‘in cis’. These results thus suggest that the widespread differences in methylation patterns between the two alleles are not due to a change in any of the diffusible elements in the cell, such as the methylation machinery, but instead likely due to local sequence changes. To put this number into perspective, we performed a similar analysis on gene expression changes in parental and hybrid strains at the meiosis arrest cells, revealing that only ~ 58% of the changes in gene expression were cis-mediated (Additional file 1: Fig. S2a,c). In this analysis we also noted that while 26% of the genes were differentially methylated between the two alleles, only 12% of the genes were differentially expressed (Additional file 1: Fig. S2b,d), perhaps hinting at stronger evolutionary constraints on gene expression than on methylatability.

To unravel the basis for cis-mediated evolution of m6A, we hypothesized that differences in methylation patterns between the two species might be a consequence of disruption of methylation consensus motifs. For this analysis we considered disruptions in the preferred sequence motif at positions -4, -2, -1, + 1, and + 4 with respect to the methylated adenosine, based on previous characterization of the preferred sequence motifs at these positions [7]. We then divided the originally identified sites into four groups, classified based on whether they were differentially modified or not, and whether they had a substitution disrupting the consensus motif or not. Examples for the various types of sites are plotted in Fig. 2c-f. Indeed, we found that in 64% of the differentially methylated sites unique to *S. cerevisiae* (DM-cer), the methylation consensus motif was disrupted in *S. paradoxus*; Similarly, in 70% of the differentially methylated sites unique to *S. paradoxus* (DM-par), the methylation motif was disrupted in *S. cerevisiae*. Conversely, among the sites with conserved m6A levels between the two species, a motif disruption was present only in 32% of the cases (Fig. 2a). Indeed, the differences in mutation patterns between the ‘differentially-modified’ sites and the ‘invariable’ ones was sufficient to recapitulate the preference for a G at positions -1 and -2, the preference for an A at position -4 and for a U at position + 4 (Fig. 2g and Additional file 1: Fig. S3), consistent with previous associative analyses. Thus, differences in m6A distribution at a sizable fraction of m6A sites is likely attributable to mutations that either give rise to birth or loss of a new m6A consensus motif.

The availability of two distinct alleles in the single hybrid strain can be conceptualized as a systematic sequence perturbation experiment, allowing the identification of the functional requirements for methylation de-novo. Accordingly, for each differentially methylated site harboring a mismatch between *S. paradoxus* and *S. cerevisiae*, we calculated the frequency of each mutation from any base into any other base for each of the nucleotides centered around the methylation motif, reasoning that such mutations are particularly disruptive. These frequencies were normalized by their counterparts in the m6A conserved set of sites, with the rationale that these should reflect a baseline of changes that do not impact m6A formation (Fig. 2g and Additional file 1: Fig. S3). Using this purely evolutionary analysis, we were able to recapitulate the preference for a G at positions -1 and -2, the preference for an A at position -4 and for a U at position + 4, consistent with previous associative analyses.

### RNA secondary structure shapes the methylation landscape in yeast

**Fig. 3:**
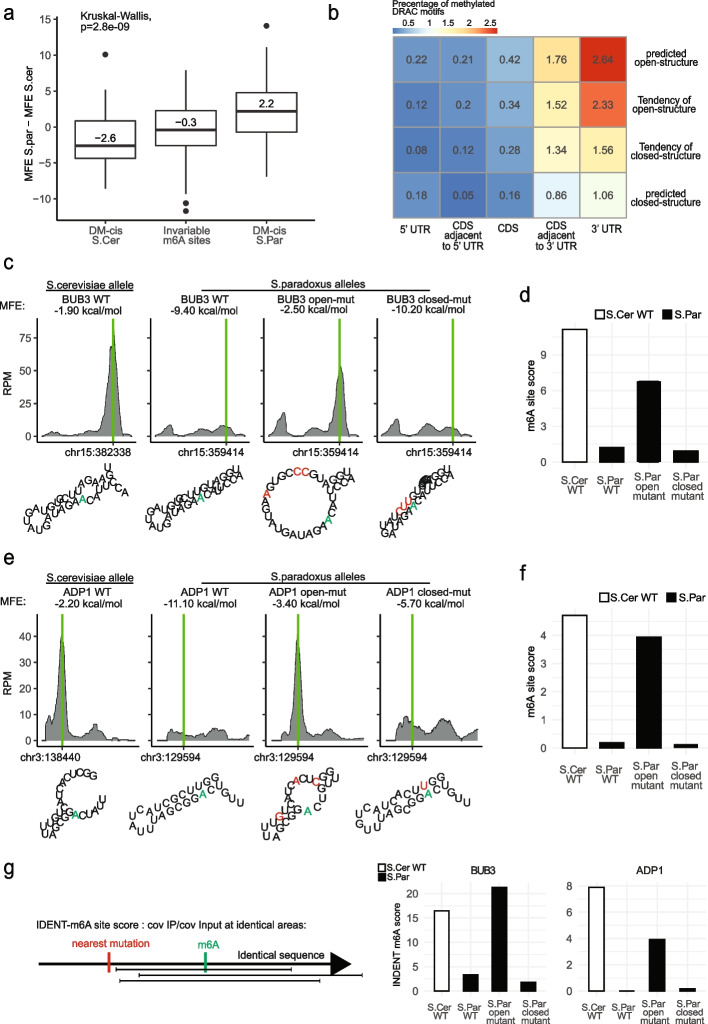
Local secondary structure around the consensus DRAC motif impedes methylation. **a** Boxplot displaying the difference in the minimum free energies values (MFE) for predicted structures between S. cer and S. par in the Invariable m6A sites (both alleles are methylated) and DM-cis sites. The prediction is made for 61-nt long window around the methylated adenosine. Sites exclusive to S. par are more structured in S. cer, whereas ones exclusive to S.cer are more structured in S. par. Sites with no variation in methylation don’t show differences in predicted structure between the alleles. To eliminate the effect of sequence on methylation and to increase the prediction tool accuracy, only sites without motif-damaging mutations and at least one allele with a predicted solid structure (MFE < -8 kcal/mol) are displayed. The whiskers correspond to the value no further than 1.5 × the interquartile range (*n* = 360). **b** mRNA secondary structure affects methylation independently of relative position within the gene. The percentages of methylated DRAC motifs are displayed along the transcriptome, binned along two dimensions: relative localization and propensity for structure. Relative localization binning was conducted on the basis of five bins: 5’ UTR, within the first 10% of CDS (CDS Adjacent to 5’ UTR), CDS, within the last 10% of CDS (CDS Adjacent to 3’ UTR), and 3’ UTR. The spectrum of MFEs was divided into four quartiles, which were used for binning sites based on structural predictions. **c** Validation of causal role on methylation played by secondary structure, based on CRISPR-based perturbations conducted in the vicinity of an m6A site in the bub3 gene. For all WT and mutant sequences, m6A-seq2 IP coverage plots are shown on the top, with green bars indicating the m6A locus and its homologous locus in the counterpart allele. The bottom set of panels depicts RNAfold structural predictions around the DRAC motif. The DRAC motif is embedded within a strong stem in the non-methylated allele (S. par WT, left panel) but not in the methylated one (S.cer WT, center-left panel). Mutating three nucleotides (depicted in red), all residing within a distance > 12 nt from the DRAC motif in S.par, led to a predicted open structure (center-right panel), and led to methylation of the DRAC motif in the S.par allele. Re-introducing a stem structure via additional compensatory mutations (depicted in red) led to elimination of the m6A signal (right panel). The methylated adenosine is depicted in green across all strains. **d** Quantification of m6A site score for the alleles depicted in (**c**). **e–f** similar analysis (as in **c**-**d**) for a site detected in the ADP1 gene in S.cer. **g** m6A IDENT-score for the m6A sites at BUB3 and ADP1. The read coverage in both IP and Input samples only considers sequencing reads that come from identical RNA fragments between the WT strain and the two mutated strains in (**c**)

While in up to 70% of the cases, differences in m6A across alleles could be attributed to changes in sequence motif, in ~ 30% of the cases, an m6A site present on one allele was abolished in the other, despite the methylation consensus motif remaining intact. We hypothesized that such changes might be due to changes in mRNA secondary structure. To explore this, we used viennaRNA package (RNAfold) to calculate the predicted minimal free energy (MFE) of the secondary structure along a 61-nt window centered around the methylation site across each of the two alleles, focusing only on the subset of sites with intact methylation motifs. Remarkably, we found that sites exclusively present in the *S. cerevisiae* allele were predicted to be substantially less structured in *S. cerevisiae* than in *S. paradoxus*. Conversely, sites present exclusively in *S. paradoxus* were predicted to be substantially less structured in *S. paradoxus* than in *S. cerevisiae*. Invariable m6A sites showed no difference in the predicted secondary structure between the two alleles (Fig. 3a). This analysis was robust to the window-size for which we predicted the secondary structure (Additional file 1: Fig. S4). These results thus suggest that the basis for the differential methylation among the group of sites with a maintained sequence motif is modulation of local secondary structure, whereby loss of secondary structure is associated with a gain in methylation. Furthermore, even when examining the subset of m6A sites in which the motif had been altered between homologous loci, the same trend (albeit at lower magnitudes) was observed (Additional file 1: Fig. S5), suggesting that even within this group a subset of the differences could be due to changes in structure, rather than in sequence, as well.

Methylation is highly biased towards gene ends in yeast, as in mammalian systems. The mechanism underlying this bias in yeast is not known, as the m6A consensus motif does not display such a bias. We thus sought to assess the relationship between 3’ bias and propensity for secondary structure. Interestingly, we found that mRNA secondary structure was conditionally independent of 3’ bias: sites in the proximity of the 3’ termini were more biased to undergo methylation independently of structure, and sites with more relaxed predicted secondary structure were more biased to undergo methylation independently of relative position (Fig. 3b).

To dissect whether the association between structure and m6A reflected a causal relationship between the two, we selected eight differentially methylated sites, all of which methylated in the allele with reduced secondary structure but unmethylated in the structured allele (see Methods for full criteria for site selection). We next generated a set of hybrid strains in which we used CRISPR/Cas9 to introduce point mutations designed to relax the secondary structure of the unmethylated allele and prevent the methylation motif from being in a stem (‘open-mut’). We also designed an additional set of strains that comprised both these mutations as well as an additional compensatory set of mutations designed to generate a stem structure in the region harboring the methylation consensus motif (‘closed-mut’). Examples of the design of two sites are illustrated in Fig. 3c, 3e (bottom). M6A-seq2 was applied to mRNA isolated from all those yeast hybrid strains, following induction of synchronous meiosis. In line with our prediction, in six of the eight tested cases, the mutations relaxing the secondary structure were sufficient to give rise to methylation in the allele that had previously not undergone methylation. Moreover, in five of the six cases in which we ‘created’ an m6A site by relaxing the secondary structure surrounding it, the compensatory ‘close-mut’ mutations that we had designed abrogated methylation at this site (Fig. 3c-f and Additional file 1: Fig. S6). Thus, these findings establish a causal role for mRNA secondary structure in determining m6A levels and demonstrate that not only can the presence of structure prevent a site from undergoing methylation, the absence of a structure can be sufficient for allowing a methylation consensus sequence to undergo methylation. These results thus establish a mechanism via which sequence disruptions at a distance from the methylation site can impact methylation levels.

We were concerned that the lack of m6A signal in the more structured allele might not be a consequence of absence of m6A, but instead reflect a potential inability of the antibody to enrich for m6A due to the double-stranded context. To rule out this possibility we performed a more detailed analysis of the m6A-seq2 data, leveraging the fact that m6A-seq2 captures the precise beginning and end of the full RNA fragments that are subjected to m6A immunoprecipitation. In three of the eight sets of sites that we had subjected to perturbations, the mutations we had introduced to open or close secondary structures were either exclusively upstream or exclusively downstream of the methylation site. This allowed us to perform an analysis of the m6A-seq2 data, considering only reads originating from RNA fragments that are shared between the alleles. For example, if mutations were introduced only upstream of a methylated site, we only considered the reads (in both IP and input experiments) including the methylation site but beginning downstream of these mutations. By doing so, the IP/Input enrichment calculation is based on exactly the same RNA sequence and hence also of associated predicted structure. Reassuringly, the results of this analysis were highly consistent with the above analysis relying on all reads, to a large extent ruling out the possibility that the lack of an m6A signal was due to a technical inability of the antibody to recognize it (Fig. 3g and Additional file 1: Fig. S6b).

### Perturbations of cis-regulatory elements are the primary determinants guiding m6A evolution in mammals

**Fig. 4:**
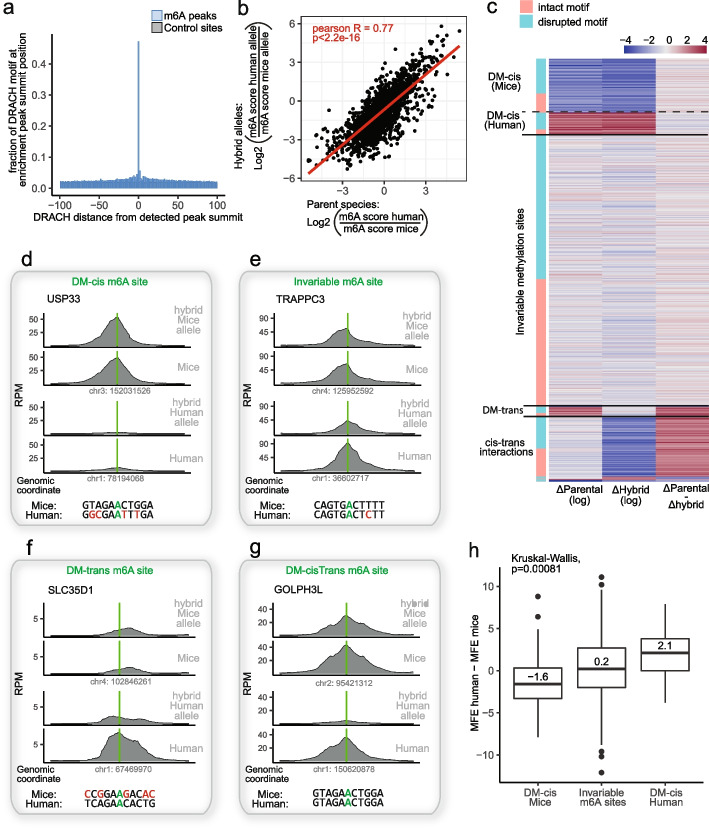
Dissection of determinants of m6A evolution in mammalian cells. **a** The relative frequency of DRACH motifs over a 200-nt window centered around the detected m6A peak summits in mammalian samples (blue), in comparison to randomly sampled regions across the same genes (gray). **b** Log2 fold changes in m6A levels between human and mouse alleles in monochromosomal hybrids (Y axis) as a function of log2 fold-changes in pseudo-parental counterparts across 2016 sites on human chromosomes 1, 2 and 3 and corresponding mouse alleles. Shown is Pearson R. **c** Classification of the detected m6A sites in mammals into differentially methylated sites in cis (DM-cis), trans (DM-trans), cis–trans interaction sites, or into invariable sites as in Fig. 2a. Δparental indicates the log2(human m6A scores)—log2(m6A scores at homologous locus at mice), while Δhybrid indicates the corresponding value between the two alleles in the cross-species hybrid. **d-g** Sequence coverage plots of m6A-seq2 IP for mice, humans, and their corresponding in the hybrids. Shown are examples of the four main regulatory determinant types of m6A sites identified in mammals: DM-cis (**d**), Invariable (**e**), cis–trans interaction (**f**), and DM-trans m6Asites (**g**). The green bars indicate the position of the detected m6A and its homologous locus on the other species, as in Fig. 2b-e. The 13-nt sequence in each of the two species/alleles are shown on the bottom, indicating the methylated adenosine (green), a motif-damaging mutation (red), or another m6A DRACH motif (green). **h** Boxplot displaying the difference in the predicted MFE between humans and mice in the two main groups of sites: invariable m6A sites and DM-cis sites. The prediction is made for 61 nt window around the methylated adenosine. Sites exclusive to humans are more structured in mice, and those exclusive to mice are more structured in humans. As in yeast, values are shown only for sites without motif-damaging mutation and at least one allele with a predicted solid structure (MFE < -8 kcal/mol) are displayed (*n* = 431)

Our results thus far establish that in yeast, m6A evolution is driven nearly entirely in cis, via alterations in the m6A consensus motif and in local secondary structure. We next sought to assess whether the same principles were true also in mammalian evolution. To address these questions, we made use of mouse-human chimeras. While full human-mouse hybrid cells are not available, the human monochromosome hybrid cell panel consists of 23 mouse A9 cell lines, each containing a different intact human chromosome generated via microcell-mediated chromosome transfer [33]. M6A-seq2 was applied to duplicates of three different cell lines from the panel, each containing a different human chromosome (chromosomes I, II, and III), a total of six samples. In the absence of true ‘parental’ cells for these hybrids, we employed m6A-seq2 on human BJ cells and mouse 3T3 cell lines serving as surrogate ‘parental’ species. As in yeast, we obtained high-resolution maps of m6A peaks (Fig. 4a and Additional file 1: Fig. S7), allowing the identification of 16,826 peaks, whereby in roughly 50% of the cases the peak summit coincided precisely with the center of the DRACH motif.

Qualitatively, this analysis yielded results reminiscent of the ones observed in yeast. Generally, differences between the human and mouse ‘parental’ cells were similar to the ones observed in the hybrid, indicative of cis-driven evolution (Fig. 4b,c and Additional file 1: Fig. S8). Moreover, as in yeast, the majority of sites fell into either a ‘DM-cis’ or ‘invariable’ cluster, with a very limited number of m6A sites that show trans evolution between the species (examples in Fig. 4d-f). Quantitatively, however, this analysis displayed some differences with respect to the one conducted in yeast. First, a considerably larger fraction of sites (64% vs. 45% in yeast) was invariable in the human-mice hybrid (despite higher genetic diversity) in comparison to the yeast hybrid. Second, in contrast to yeast, in the human-mouse hybrid, a larger fraction of the defined m6A sites (12%) fell into the cis–trans interactions (examples in Fig. 4g), which display differences in methylation between the two alleles in the hybrid (hence classified as ‘cis’, Fig. 4c middle column) but whereby these differences in the hybrids are not maintained in the pseudo-parental cells (hence ‘trans’, Fig. 4c right column). Inspection of the directionality of these changes revealed that in the vast majority, such ‘cis–trans’ classified sites were lowly methylated in the human allele in the hybrid strain in comparison to the mouse counterpart, which was not mirrored in the parental species. To explore the basis for these differences, we examined expression levels of the genes harboring these peaks in the parental and hybrid strains. We noted that the vast majority of peaks within the cis–trans cluster of peaks (roughly 70%) resided within genes that were disproportionately more lowly expressed in the human alleles in the hybrids than in the parental strains (Additional file 1: Fig. S9a-b). In contrast, their corresponding mice alleles are expressed at anticipated levels (Additional file 1: Fig. S9c-d). Indeed, a more formal examination of this, through the lens of cis/trans regulation of gene expression, revealed that roughly 65% of these cis–trans m6A sites resided within genes classified as being subject to cis–trans regulation at the expression level, in contrast to invariable or DM-cis peaks, where only 28% and 20%, respectively, resided in cis–trans regulated genes (Additional file 1: Fig. S10). Thus, the discrepant m6A levels between the two alleles of the hybrid are associated with discrepancy gene expression levels from the two alleles, which could be due to biological considerations (e.g. differential mRNA metabolism coupled to methylation) or technical ones [34]. Across all sites, the correlation between the delta-m6A profiles in parental species vs hybrids was *R* = 0.77. This is considerably lower than the observed correlation in yeast (*R* = 0.96), yet still supportive of changes in ‘cis’ being the primary source of m6A evolution. This lower correlation is driven, in part, by the ‘DM-trans’ and ‘cis–trans’ clusters, and may also reflect the fact that in contrast to yeast in which we could make use of the actual parental strains, in the human-mouse hybrids such perfectly matched parents are not available, likely injecting technical noise into the measurements. As in yeast, comparing the sequence mismatch profiles between the DM-cis and the invariable clusters allowed us to de-novo infer the preferred m6A consensus motif (Additional file 1: Fig. S11).

Next, we considered the factors driving cis evolution in the human-mouse hybrids. 77% of the DM-cis sites in the hybrid were associated with mutations disrupting the m6A consensus motif, likely accounting for the changes in methylation status at the detected sites. An analysis of the DM-cis sites lacking motif-damaging sequence changes between the species at the extended m6A motif revealed that the methylated adenosine tended to be in a more relaxed secondary structure compared to its homologous locus that did not undergo methylation, consistent with the above results in yeast. In contrast, control sites that were equally methylated between the alleles also showed no changes in predicted secondary structures (Fig. 4h). Thus, the role of secondary structure in determining methylation susceptibility appears to be conserved also in mammalian evolution.

**Fig. 5:**
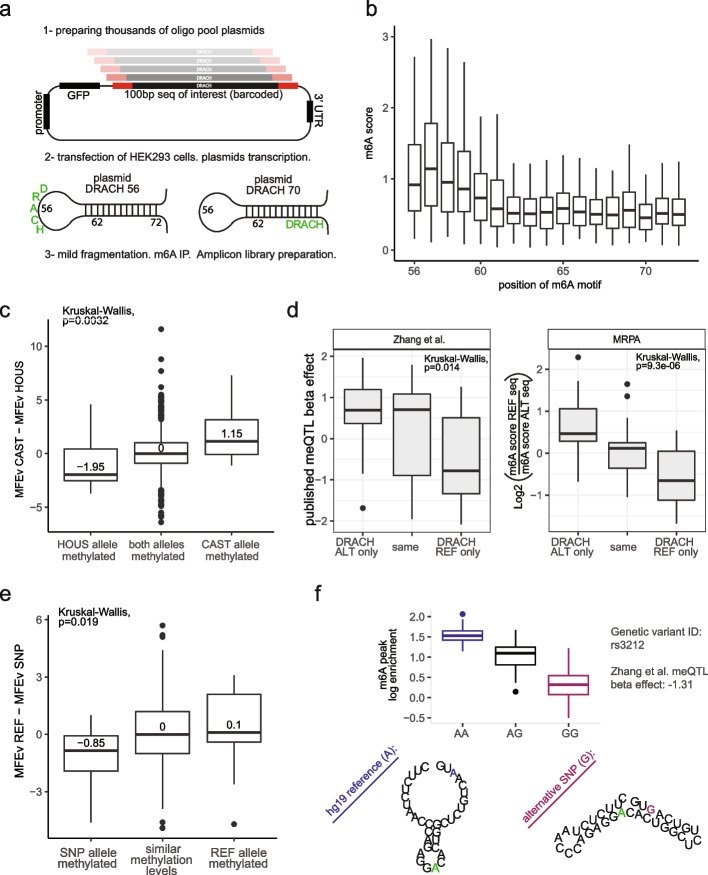
Sequence and RNA secondary structure around methylation consensus motifs determine variability in m6A levels across mammalian individuals of the same species. **a** Scheme depicting the design of a massive parallel reporter assay aiming to establish the impact of secondary structure on m6A formation. For each of 120 m6A sites, we designed a set of oligos in which we systematically shifted the relative position of the methylated DRACH motif from a fully open loop (methylated adenosine at position 56) into the stem (starting from position 62). **b** Distribution of m6A enrichment (IP/input) levels as a function of the relative position along the sequence (and secondary structure), depicting a gradual decrease in m6A levels as the consensus motif is shifted from positions in the loop to positions in the stem. **c** Boxplot displaying the differences in the predicted MFE for sites detected either exclusively in the castaneous strain, only in house mice, or in both, as in Fig. 4h (n = 969). **d** *Left*: distribution of beta values, obtained from Zhang et al. (22), across 101 ‘proximal’ m6A-QTLs, binned based on whether the m6A QTLs lead to the formation of a site exclusively at the reference allele, alternative allele or does not impact methylation status. The beta represents the correlation between the SNP and allele-specific methylation scores of 60 YRI human individuals, with negative beta indicating that the hg19 reference genome variant is methylated, while positive beta indicates that the alternative variant leads to higher methylation levels at the locus. *Right*: for the same 97 m6A sites, displayed are the m6A scores ratio for the synthesized 101-nt window sequence centered around the methylated adenosine, derived from either the reference or the alternative sequence. **e** Boxplot as in (c) for 60 different human individuals as published in Zhang et al. Methylation sites exclusive to the reference allele were defined as ones with beta > 1, whereas ones exclusive to the alternative allele were defined as beta < -1. Betas within the range of -1 to 1 were considered to display similar methylation patterns between the published genetic variants. Only sites without motif-damaging SNP and at least one allele with a predicted solid structure (MFE < -8 kcal/mol) are displayed (*n* = 152). **f** An example of an m6A-QTL site that was found by Zhang et al. (22), which did not harbor an m6A consensus motif disrupting SNP. The top panel is a boxplot representing the m6A scores for each of the population genotypes (*n* = 60 independent samples). Boxplots correspond to the median, Q1 and Q3, whiskers mark Q1-1.5 IQR and Q3 + 1.5 IQR. The lower panel illustrates the predicted MFE secondary structure around the DRACH motif nearest to the m6A-QTL. The m6A-QTL sequence variation is marked in blue on the reference genome and in red in the alternative allele

To systematically confirm the importance of secondary structure in controlling methylation levels in a mammalian system, we designed a massively parallel reporter assay to interrogate the status of 2040 sequences, in which we varied the relative position of m6A sites within a stem-loop (Fig. 5a-1). Specifically, in this assay, we selected 100 human m6A sites. For each site, we extracted a 101-nt window centered around the methylated site, and manipulated these sequences to contain a 21-nucleotide perfect stem sequence with an 11-nucleotide loop, and systematically shifted the relative position of the DRACH motif from the middle of the loop (position 56) to the middle of the stem (position 72) (Fig. 5a-2). The set of oligos were synthesized and cloned into the 3’UTR of an Snrpn-GFP plasmid. The pool of plasmids was transfected into Hek293T cells as previously reported [35]. Application of m6A-seq2 revealed that the DRACH motif is methylated to its highest levels when it is in the center of the loop, in which the entire DRACH motif is open-stranded. Gradual shifting of the DRACH motif from the loop into the stem led to a continuous drop in methylation levels, and minimal methylation levels were observed upon shifting the entire DRACH motif into a stem (Fig. 5b), consistent with our results in the hybrids.

### Clustered m6A sites lead to robust m6A peaks despite abolishment of motifs

The above analyses provided insight into the subset of differentially methylated sites (DM-cis) in which methylation was modulated despite the intactness of an m6A consensus motif. Unexpectedly, both yeast and mammalian hybrids comprised a set of sites with opposite characteristics, namely ones in which m6A levels did not change (forming part of the ‘invariable’ group), despite the m6A consensus motif being disrupted in one of the two alleles (Figs. 2a and 4c). In yeast, ~ 32% of the sites in the ‘invariable’ group had a mutated consensus motif, and in mammals 53%. We hypothesized that the persistence of antibody-mediated enrichment at these loci in the allele in which the m6A motif was disrupted might stem from methylation at adjacent, potentially newly-formed, m6A consensus motifs. Under this scenario, an enrichment in m6A consensus motifs would be expected to be present in the allele harboring the mutated m6A consensus motif. To examine this hypothesis, we first counted the number of newly formed (‘emerged’) m6A sequence motifs at the homologous loci of an m6A site. In both human and yeast there was a substantial enrichment of ‘emerged’ m6A consensus motifs in the 61-nt window surrounding the mutated methylation site, both in comparison to other sites in the ‘invariable’ group where the motif had not undergone disruption, and in comparison to sites in the DM-cis groups (Additional file 1: Fig. S12a,b). To obtain experimental confirmation that the adjacent sites were, indeed, methylated, we leveraged recently produced highly-sensitive GLORI-based measurements of m6A at single-nucleotide resolution in human and mice cells. Consistent with this hypothesis, we found that (1) A window of 61 nt surrounding ‘Invariable’ sites tended to comprise more m6A sites than DM-cis sites, and (2) Sites in the ‘invariable’ group with a mutated consensus motif comprised a higher frequency of methylated m6A in their immediate surrounding than counterparts with an intact motif (Additional file 1: Fig. S12c). Thus, the persistence of an enriched signal despite the abolishment of consensus motifs stems—at least in part—from methylation at adjacent sites. The fact that this phenomenon is considerably more widespread in human than in yeast hints that the propensity for m6A sites to occur as larger clusters may be higher in humans than in yeast. Increased propensity for m6A clustering in human, in comparison to yeast, would also provide a rationale for why the relative frequency of ‘invariable’ sites is generally higher in humans than in yeasts.

To experimentally confirm that m6A signal can persist despite loss of consensus motifs due to compensatory signals from adjacent sites, we utilized a series of sequences for which we had measured m6A levels as part of a massively parallel reporter assay [6]. Specifically, we analyzed a series of 101-nt long oligos centered around methylated DRACH motifs (based on miCLIP data) (32) which had been cloned into the 3’ end of intronless GFP, downstream of a Snrpn promoter (WT). For each sequence we also cloned a point mutated counterpart, harboring a DRTCH motif instead of DRACH, which will entirely abolish methylation of the consensus motif. A comparison of the enrichment (IP/input) in the point-mutated sequences in comparison to their WT counterparts revealed that the extent of depletion of signal was inversely correlated with the number of additional DRACH motifs in close proximity (Additional file 1: Fig. S12d). These findings thus demonstrate that the overall methylation signals manifesting in a single peak can often stem from multiple, closely adjacent methylated motifs, providing a rationale for how enrichment signal can persist even upon complete loss of one of those motifs.

### Dissecting changes in m6A levels within individuals of the same species

We reasoned that the forces giving rise to the evolution of m6A across different species likely also shape differences in m6A levels between individuals of the same species. To explore this question, we first used mouse embryonic stem cells originating from mating two different mouse strains (house mouse and castaneous). The genetic background of the two strains is well characterized, and each of the two alleles in the F1 hybrids is fully phased. We performed m6A-seq2 and aligned the reads against a combined genome of both strains. We detected 10,392 m6A peaks, at a median distance of 1-nt from the nearest consensus site (Additional file 1: Fig. S13a). While the vast majority of sites (~ 99%) were conserved between species, ~ 1% of the sites were differentially methylated. Of these, ~ 20% of the sites comprised a disrupted m6A consensus motif (compared to ~ 0.5% at the ‘Invariable’ m6A sites) (Additional file 1: Fig. S13b). Examination of the remaining sites revealed that house mouse unique sites tended to be less structured in house mouse allele, whereas sites unique to castaneous tended to be less structured in castaneous allele (Fig. 5c). Thus, m6A within a species is controlled via similar mechanisms guiding m6A across species.

As a final step, we sought to address whether the same rules underlying methylation differences between different mice species also underlay differences in methylation patterns among human individuals. Exploring this is of particular interest, given their potential relevance to human disease. Previous studies have sought to systematically identify m6A quantitative trait loci (m6A-QTLs) on the basis of m6A maps produced across species from dozens of individuals. These studies identified thousands of SNPs associated with differences in m6A levels, typically at a considerable distance from the m6A sites, some of which are also linked to human diseases. However, how these SNPs impacted m6A levels remained largely unknown. To explore these questions, we re-analyzed a dataset of methylation maps produced across 60 different genotyped individuals [22], in which ~ 13 million genetic variants were associated with 20,637 m6A peaks. While the vast majority of these associations are unlikely to be causal, we speculated that a subset of them might be, in which case they might directly impact m6A formation by impacting sequence and or secondary structure. We therefore sought to identify such potentially causal SNPs. Of note, the m6A-seq data produced in this study was of substantially lower resolution than counterparts produced using our m6A-seq2 protocol (Additional file 1: Fig. S14a). Nonetheless, binning sites based on significance, it was evident that bins of increased statistical significance tended to be substantially closer to the impacted m6A site (Additional file 1: Fig. S14b), hinting at an increased propensity to be causal. We further noted that m6A-QTLs within exons tended to be substantially closer to the center of an m6A peak than intronic counterparts (Additional file 1: Fig. S14c). Based on these analyses, we filtered for QTLs at exonic m6A peaks with *p*-value < 0.01 residing within up to 50 nt from the center of the called peaks. In line with our anticipations, we found that mutations in the m6A consensus motif were substantially enriched in this subset of proximal m6A-QTLs, with 33% of them either disrupting an m6A motif present in the reference allele or giving rise to a new m6A motif in the alternative allele, in comparison to only ~ 15% at randomly selected sites or of sites with insignificant *p*-value. Newly formed m6A consensus motifs in the alternative allele (absent in the reference) were associated with positive beta values in the original study, whereas disrupted m6A alleles in the alternative allele were associated with negative beta values, all consistent with these m6A-QTLs playing a causal role in determining m6A formation.

To ensure that these motif alterations across alleles were causal, we selected 101 sites harboring a ‘proximal’ m6A-QTL with a *p*-value < 0.01 and interrogated m6A levels on both their ‘reference’ form and their ‘alternative’ form via an m6A massively parallel reporter assay (Fig. 5d). As anticipated, m6A-QTLs associated with disruption of an m6A consensus motif were indeed methylated to lower extents than their motif-harboring counterparts, whereas variants associated with the formation of a motif were methylated to higher levels than their motif-lacking counterparts, establishing that many of these m6A-QTLs are not only *associated* with m6A but instead play a *causal* role.

Having established that many of these proximal m6A-QTLs can be causal, we sought to investigate the role played by mRNA secondary structure. Focusing on proximal sites in which the m6A motif was not disrupted across individuals, we found that the motif-disrupting m6A-QTL was associated with increasing the propensity for secondary structure at the methylated site (Fig. 5e-f). The relatively weaker signal observed in this analysis, in comparison to previous analyses, likely reflects at least in part the decreased resolution of the data imposing challenges on assigning individual m6A sites to broader peaks. Collectively, these results establish two mechanisms via which m6A-QTLs can causally direct m6A levels, either by impacting the m6A consensus motif or by impacting local secondary structure.

### Dissection of evolutionary consequences of methylation changes

**Fig. 6:**
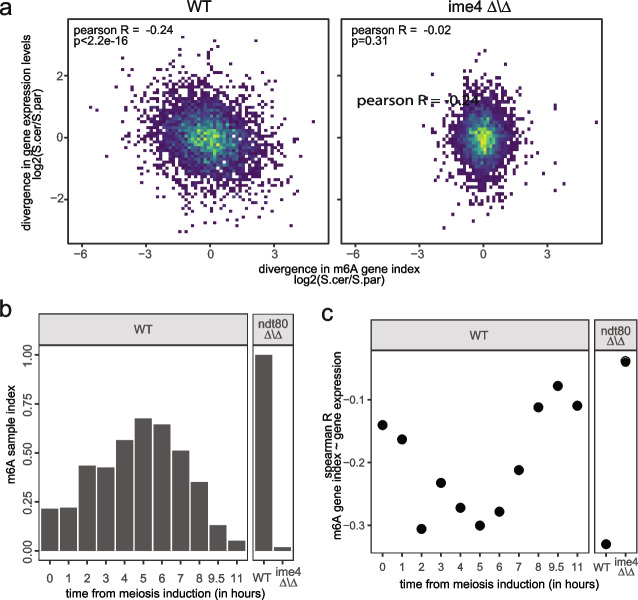
Changes in m6A levels between species are associated with coherent changes in gene expression levels. **a** The fold-change of gene methylation levels (m6A gene index) between *S. cerevisiae* and *S. paradoxus* alleles in the ndt80Δ/Δ hybrid (y-axis) depicted as a function of the fold-change in gene expression levels (measured as sample TPM) between the alleles appears at the x-axis. Higher methylation levels of an allele correlate negatively with its expression in ime4 WT (left) but not in a methylation-lacking (ime4Δ/Δ) strain, indicating abolishment of the association. **b** Quantification of m6A levels via m6A-sample index along a dense meiotic time course. Values for the ndt80Δ/Δ mutant and for ndt80Δ/Δ ime4Δ/Δ double mutants are displayed as positive and negative controls, respectively. **c** Calculation of Pearson R for agreement between differences in methylation and in expression across the two alleles (as in panels a and b), at different time points after induction of yeast meiosis in the WT hybrid strain. The anticorrelation between allele-specific methylation and allele-specific expression increases dynamically with the gaining of methylation during meiosis progression up to prophase, and again continuously lost with the decrease in methylation following prophase

Our results thus far establish that extensive differences in methylation have occurred even between two closely related species. We were next interested in dissecting the consequences of these changes. Given the well-established role of m6A in triggering mRNA decay, we were particularly interested in dissecting whether the differences in methylation between two species leads to predictable differences in the relative abundances of the genes. Indeed, we found that differences in m6A levels between the two species were inversely correlated with differences in expression levels in WT cells (*R* = -0.24), consistent with a destabilizing role played by m6A (Fig. 6a, left). In contrast, this association was abolished in ime4 KO cells (*R* = -0.02) (Fig. 6a, right). To further explore this relationship, we monitored both m6A and gene expression over a densely sampled meiosis time course in the hybrid. At each timepoint we monitored the association between differences in gene expression and differences in methylation levels. We observed that the inverse association between the two increased up to the 5 h timepoint, at which point it gradually decreased (Fig. 6c), mirroring the accumulation patterns of m6A (Fig. 6b). A similar analysis conducted on mammalian monochromosomal hybrid system similarly revealed an inverse correlation between changes in mRNA methylation and changes in gene expression (Additional file 1: Fig. S15). These results thus suggest that in both yeast and mammalian hybrids, differences in gene expression levels between species are controlled in part by differences in m6A profiles, whereby in yeast the extent of such tuning varies along meiosis as a function of m6A levels.

## Discussion

The question of whether the evolution of a cellular output is governed in cis versus in trans is a fundamental one, as its dissection points to the genetic source controlling its variation. Diverse layers of regulation have been dissected on the basis of interspecies hybrids and genome-wide association studies. These studies often pointed at cis-based regulation as a major source of difference between species, accounting for nearly 100% of the divergence in DNA methylation, and accounting for most of the variability in most types of alternative splicing and RNA deamination [36–38]. Yet, certain regulatory levels are regulated in trans. For example, trans-regulatory variation was shown to contribute extensively to differences in exon-skipping events between drosophila species [39], to changes in activity of enhancers [40], and to changes in translation efficiency between yeast species [26]. In our previous study [7], we provided evidence suggesting that a substantial portion of m6A modifications is "hard-coded" in cis, given that a linear model, primarily relying on the sequence composition within a 9 nt window centered around the methylation site, could account for approximately 33%-46% of the variability in m6A levels in yeasts and mammals, respectively. The remaining variability could, in principle, be either mediated ‘in trans’, e.g. via factors that specifically recruit m6A writers or erasers to specific targets, but potentially also ‘in cis’, via components not accounted for in our model. Our current study relies on a fundamentally distinct strategy, allowing to *formally* distinguish ‘cis’ from ‘trans’ effects, without any prior assumptions regarding the determinants governing m6A formation. Dissection of m6A via this interspecies lens now reveals that in yeast nearly 100% of the observed differences in m6A patterns can be attributed to cis determinants. In mammalian systems cis-regulated determinants accounted for approximately 60%. The remaining variability might either be technical in nature—given the absence of matching parental species and limitations in the mammalian interspecies hybrids—or might reflect non-cis regulation. One way or another, our findings provide valuable insights into the regulatory mechanisms shaping m6A differences across species, highlighting the crucial role of cis-regulatory elements in driving the evolutionary dynamics of this critical epigenetic mark, substantially higher than could be estimated based on previous studies.

Oftentimes hybrid-based studies, relying on divergence of sequence that happened to be introduced by nature rather than by rational or systematically designed sequences, do not readily lend themselves to mechanistic insights. In this case, however, the differences between alleles were highly interpretable, pointing at a strong sequence and structural signatures that, together, account for the majority of changes observed between alleles. While the role of sequence was anticipated, the substantial role of secondary structure in establishing m6A maps was more surprising. RNA secondary structure was found to inhibit m6A formation in-vitro, in experiments in which the METTL3-METTL14 heterodimer was exposed to mRNA [11]. Yet, whether secondary structure enhanced, or repressed, m6A formation in vivo was unclear, given that propensity towards stronger predicted mRNA secondary structures was shown to correlate negatively with m6A formation in some studies [7, 8], but to correlate positively in others [12, 41], and in both cases the reported effect sizes were relatively weak. Hybrids offer a substantially more powerful approach to dissect these questions than associative, genomic studies. Rather than relying on drawing associations between m6A levels and secondary structures across different sites, they allow comparing m6A measurements at one site with closely matched (yet genetically distinct) counterparts on the corresponding allele, in this sense coming close to a perturbational experiment. Our hybrid-based measurements, further supported by massively parallel reporter assays and by mutational assays, point at a fundamental role of secondary structure in modulating the propensity of m6A formation and in shaping transcriptome-wide distribution of m6A. Given that mRNA secondary structure is dynamic, and can be molded by interactions with RNA binding proteins, it is interesting to speculate that differences in mRNA secondary structure—for instance across biological trajectories or in response to environmental cues—might lead to modulation of m6A levels.

The widespread changes in m6A in cerevisiae (45% of sites) compared to mammals (22% of sites) may be due to increased propensity of m6A sites to cluster together in mammalian systems, than in yeast. Alternatively, these changes could also hint at there being decreased selective evolutionary pressure on m6A in yeast, in comparison to mammals. Such a possibility would be consistent with a previous study, reporting no evidence for selective pressure acting on methylation consensus motifs in yeast as opposed to higher pressure in mammalian systems [42], and with another study finding evidence for selective pressure acting on m6A in primate evolution [43]. The fact that in yeast methylation is restricted to meiosis, whereas in mammalian systems it is constitutively active, may provide a rationale for the increased selective pressure in mammalian systems in comparison to yeast. Nonetheless, the observation that gene-level m6A measurements are more conserved across species than site-level measurements (Additional file 1: Fig. S2a and Additional file 1: Fig. S9a) may suggest that changes in methylation at individual sites can be buffered, at the gene level, via compensatory changes. Such findings would be consistent also with an evolutionary analysis, indicating stronger evidence for selection at the gene level [42]. Nonetheless, it is critical to emphasize that our work was not designed, nor is it powered, to allow quantifying evolutionary forces acting on m6A, for which a substantially more dense phyletic sampling would be required. Furthermore, we also cannot rule out that the more conserved patterns at the gene level, than at the site level, are in part also due to the fact that variability of the mean (in this case: m6A-GI over entire genes, reflecting signal originating from multiple methylation events) is lower than variability of individuals (in this case: individual sites). For example, under a scenario wherein in each species DRAC motifs are selected for methylation randomly at a fixed propensity (e.g. 5% of DRAC motifs are methylated), substantial fluctuations between two alleles would be evident at the level of individual sites, but such changes would appear buffered at the gene level.

Our study suffers from several limitations, many of which are linked to limitations of m6A-seq2. First, in interpreting differences between alleles we conservatively opted to analyze the data in a binary (qualitative) way, with sites being decreed either present or absent. In practice, differences can oftentimes likely also be quantitative. Second, while our experimental and computational pipeline allows us to obtain a nearly single-nucleotide resolution view for many of the sites, it is not inherently single-nucleotide, and as such we anticipate that in a minority of cases we may have wrongly assigned a motif to a peak. This limitation in resolution is even more pronounced in the context of interpreting previously established m6A-QTL sites, on the basis of an m6A-seq dataset with substantially lower resolution. The difficulty in precisely pinpointing the m6A site in this dataset renders it challenging to dissect the mechanism via which variability in sequence impacts methylation. Finally, the sensitivity of m6A-seq2—in particular when combined with stringent peak detection—is limited, as was concluded based on comparison with an antibody-independent method [7]. Recently developed improved techniques for mapping m6A in a quantitative manner at single-nucleotide resolution [30, 44] will allow to revisit m6A evolution using a more precise, comprehensive and quantitative lens.

## Conclusions

In this study we dissect the forces governing m6A evolution and variation, revealing that both between yeast and mammals m6A evolution is primarily governed by changes in sequence rather than by changes in the cellular environment. By carefully dissecting the differences in m6A, we identify two mechanisms underlying the changes: changes in the m6A consensus sequence and changes in the target site secondary structure. We establish the causality of these changes and their relevance also to changes in m6A levels between individuals of the same species. Our study establishes an understanding of the cis-mediated determinants and underlying mechanisms of action guiding m6A evolution between species, and variability within species, and paves the path to dissecting the roles played by m6A altering genetic variants in health and in disease.

## Methods

### Yeast strains and meiosis

The *S.cerevisiae* yeast strains used in this work were derived from the sporulation-proficient SK1 strain background [7]. The *S. paradoxus* yeast strains used in this study were purchased from NCYC, National Collection of Yeast Cultures (Quadram Institute Bioscience, Norwich Research Park, Norwich, UK). Deletion of ndt80 and ime4 genes (generating ndt80Δ/Δ and ime4Δ/Δ strains) were performed on the haploid cells with different MX cassettes: hphMX, natMX or kanMX cassettes giving rise to different types of selections. We then mated the haploids and grew the mated cells on double-selection plates (Additional file 2: Table S1).

#### Generating the yeast hybrid strain

To create the yeast hybrids, we mated the SK1 mat-a strain with the YPS138 mat-alpha strain on YPD plates in all genetic backgrounds (WT, ndt80Δ/Δ, ndt80Δ/Δ + ime4Δ/Δ). After 24 h, we streaked the cells on double-selected plates and isolated a few colonies. We then confirmed the successfully mated colonies via sanger sequencing of relevant areas containing substantial sequence divergence. The relevant genomic regions were PCR-amplified from the hybrid genome.

#### CRISPR cas9 perturbation

Our criteria for site selection involved identifying regions with differential methylation patterns in homologous sequences, exhibiting a predicted reduction in secondary structure at the methylated species. In selecting sites, we loosely considered the following factors: (1) Prioritizing sites with clear-cut differences between the two species, (2) Giving preference to m6A sites without additional peaks in close proximity in both species to prevent signal leakage, (3) Focusing on sites predicted to possess a well-defined and interpretable structure, such as a stem loop, (4) Requiring that in the predicted structure, the methylated adenosine and its immediate neighbors be paired with other nucleotides in the unmodified allele, and (5) Prioritizing sites where minimal genetic intervention (via nucleotide substitutions) could result in a maximal alteration of the predicted secondary structure. CRISPR transformations were performed using the bRA89 backbone plasmid and the pJH2972 backbone plasmid, encoding Cas9, the target-specific guide-RNA, and Hygromycin resistance or URA3 gene, respectively. We designed the appropriate mutations based on RNAfold prediction for both 61nt and 51 nt windows centered around the methylated adenosine (The latter is displayed in Figs. 3 and 5). The designed guide RNAs were ligated into the pre-cut vectors as detailed [45]. The ligated vector was transformed into Escherichia coli for propagation and the plasmid inserts were verified with PCR and purified with MiniPrep Kit (NEB, catalog no. T1010S) (Additional file 2: Table S2). We then transformed the plasmid into *S.cerevisiae* and *S. paradoxus* haploids to generate the mutant haploids. Next, we mated the mutant strain with an opposite haploid strain of the other species. In total, we generated 14 additional mutants, two additional hybrids for each site (as for the two m6A sites presented in Fig. 3).

#### Growth and medium conditions

To induce synchronous meiosis in all strains, cells were grown for 24 h in 2% YPD (1% yeast extract, 2% peptone, 2% dextrose) at 30 °C 230 r.p.m and then diluted to OD600 = 0.3 in 4% YPD (1% yeast extract, 2% peptone, 4% dextrose) at 30 °C 230 r.p.m. We then diluted the cells to OD600 = 0.2 in BYTA (1% yeast extract, 2% tryptone, 1% potassium acetate, 50 mM potassium phthalate) and let it grow for additional 16 h at 30 °C at 230 r.p.m. Cells were washed three times with water, resuspended in SPO medium (0.3% potassium acetate) at OD600 = 2.0, and incubated at 30 °C for 6.5 h at 190 r.p.m before collection.

### Mammals cell lines and cell culture

Human monochromosome hybrid cells JCRB2201, JCRB2202 and JCRB2203 were purchased from the Japanese Collection of Research Bioresources (JCRB), and cultured in DMEM (GIBCO) supplemented with 10% fetal bovine serum (FBS) and 0.8 mg/ml G418(Gibco,79N9555) [46]. mESCs, BJ, and NIH 3T3 cells were cultured in DMEM (GIBCO) supplemented with 10% FBS and 1% Penicillin and Streptomycin. Cells were routinely tested for mycoplasma contamination and were not authenticated.

### RNA preparation

Yeast total RNA samples were prepared by adjusting previously published protocols [8, 47]. After fast freezing the yeast cells in liquid nitrogen, yeast cells were resuspended in equal amounts of phenol:Chloroform:Isoamyl alcohol (Sigma Aldrich), buffer AE (50 mM sodium acetate, 10 mM EDTA 1% SDS), and glass beads in Eppendorf tube. The tube was vortexed for 15 min in a bullet blender, heated to 65 °C for 30 min, followed by another round of 5 min vortex and 30 min heating to 65 °C. Samples were placed on ice for 5 min and centrifuged for 10 min (12000 g, 4 °C). The supernatant was isolated, re-extracted with phenol:Chloroform:Isoamyl alcohol, and precipitated with sodium acetate and isopropanol.

For all types of mammalian cell lines, total RNA was extracted with BIO TRI RNA reagent (Bio-lab). Enrichment for mRNA was done by two rounds of poly-A selection using Oligo(dT) beads (Dynabeads mRNA DIRECT) in both total RNAs from yeast and mammals.

### m6A-IP

m6A immunoprecipitation and NGS library preparation were prepared based on the m6A-seq2 protocol [5]. In the first round of IP we used Dynabeads protein G beads (Invitrogen) and 3.5 µl of anti-m6A antibody (Synaptic Systems, poly-clonal antibody, Rabbit, in H2O), both as in the original protocol. In the second round of IP we used Dynabeads protein A beads (Invitrogen) and 6.8uL of a different anti-m6A antibody (Cell Signaling D9D9W).

### m6A enrichment data analysis

The paired-end reads were demultiplexed into individual samples and aligned to the appropriate genome using STAR 2.5.3a [48]. All the yeast samples were aligned to a combined genomic fasta file containing all S.cer and S.par chromosomes. The monochromosomal hybrid samples, were aligned to a combined mm9-hg19 fasta file (all mm9 chromosomes concatenated with hg19 chr1, chr2 and chr3). The CAST-HOUS mice hybrid samples were aligned to a combined fasta file containing both strain's chromosomes. Read coverages were obtained using the R package txtools (https://github.com/AngelCampos/txtools) [49], and normalized to the total number of reads in the pool.

#### De novo* m6A peak calling*

Peak calling for de novo detection of m6A sites was done similarly to our previous study [5], relying on score-1 (quantifying IP/input levels) and score 2 (quantifying enrichment in IP at a given locus in comparison to the median signal within a gene). Briefly, we assessed the m6A site score2 (5) for each transcriptomic position. We calculated this score by dividing the mean coverage in a 51nt window centered around the position by the median coverage of the gene in the IP-treated sample. Subsequently, we identified enrichment windows by selecting consecutive stretches that were longer than 15 nucleotides, with an assigned m6A score2 > 4 at each position. We determined a similar m6A score2 for the input sample and defined a winScore, which we calculated as the ratio of both scores. We filtered out windows with winScore < 2 in any of the replicates. We then calculated the aggregate coverage density for each window by aggregating the coverage densities of all replicates. We identified the peak summit and then searched for the nearest DRAC motif (In mammals, DRACH) which we considered as the putative methylated site. We assigned an m6A score1 for each detected site as a ratio of the mean of the m6A-IP coverage per base in an 81-nt window centered around the annotated m6A site divided by the corresponding value in the input sample. We requested that the m6A score1 will be > 3. In the yeast samples in which we also had methyltransferase KO strains, we demanded that the detected enrichment window score ratio between WT and KO in one of the species will be > 3. We also requested that the calculated student T-test P-value between the WT and the KO be smaller than 0.05 for either m6A score1 or msA score2. Only m6A score1 (a measure of IP/input signal) was used across the manuscript to quantify m6A levels, and referred to as ‘m6A enrichment’ (Additional file 3: Tables S3-S5 and Additional file 4: Tables S6-S8).

#### Motif-disrupting mutations

Motif-damaging mutations were defined based on our previous work [7], and comprised the following: Position -4: A- > C/G/U. Position -2: G- > A/C/U, A- > C/U, U- > C. Position -1: G- > A/C/U, A- > C/U. Position 0: A- > C/G/U. Position + 1: C- > A/G/U. Position + 4: U- > A/C/G. In mammalian samples, position + 2: A/C/U- > G.

#### m6A sample index

The m6A-SI for a sample m6A quantification estimate was defined as the ratio of the sum of normalized coverage reads in the IP-treated dataset divided by the corresponding number in the input dataset, across all species-specific identified m6a peaks, as described [5].

#### m6A gene index

The m6A-GI is calculated as the ratio between the number of reads aligned to the entire gene in the IP-treated sample normalized by the corresponding reads number in the input sample.

### Randomly sampling control sites

To generate control sites for the specificity plots in Figs. 1c and 4a, Additional file 1: Fig. S7, S13a, and S14a, we randomly selected positions from the same genes in which the m6A peaks were identified. For the analysis presented in Additional file 1: Fig. S13a and Additional file 1: S13b, we followed a similar approach and chose random DRAC(S12a) or random DRACH(S12b) sites from the same genes as any detected m6A site.

### Homology detection

#### Yeast samples

To find the corresponding locus for each detected position in the corresponding species, we used two independent tools: emboss WATER [50] and BLAT [51]. We defined homologous genes based on the yeast population reference panel (https://yjx1217.github.io/Yeast_PacBio_2016/data/) [52]. Using the emboss WATER tool, we aligned each gene fasta file to its homolog gene with the default parameters (-gapopen 10 -gapextend 0.5). We used an in-house script to locate the homologous locus from the generated output (.water file). To expand the pool of homologous m6A sites, we utilized BLAT to create MAF files comparing the gene that contained the detected m6A site from each species to the genome of the other species (with the command blat genome_speciesA.fa m6AdetectedGene_speciesB.fa -out = maf). We then used an in-house script to locate the corresponding locus based on the output file. We filtered out a small subset of cases (0.3%) in which the emboss WATER and BLAT based approaches did not converge on the same site.

#### Mice-Human samples

To find the homology coordinates between mice mm9 and human hg19, we used available multiZ MAF files sources at the UCSC browser [53]. For sites detected in human hg19, we used the Multiz alignment of 46 vertebrate genomes to get its mm9 corresponding coordinate. For sites seen in mm9, we used the Multiz alignment of 30 vertebrate genomes to get the hg18 coordinate. We then used an in-house R script (https://github.com/AngelCampos/txtools) to generate chain files that can be utilized by the Liftover tool [54] to convert the genomic coordinates of hg18 to hg19.

#### mice hybrid

Chain files to convert between CAST fasta genome to HOUS fasta genome were generated using an in-house R script (https://github.com/AngelCampos/txtools). By using the liftover tool on those chain files, we assigned each detected m6A site the homologous coordinate in the other species.

### Massively parallel reporter assay

The short oligos described in this study (Fig. 5, Additional file 1: Fig. S13) were designed as part of a larger pool of sequences that was probed and characterized in [6]. These sequences were synthesized by Twist Biosciences and cloned into an SNRPN-GFP plasmid, expressed within Hek293T cells and subjected to m6A-seq as detailed in [6]. The design of short oligos relating to Additional file 1: Fig. S13 is detailed in [6]. The ‘structural mutation’ series (Fig. 5a-b) is based on 120 high-confidence human m6A sites that were reproducibly detected in different miClip studies. Each sequence was 101 basepairs long, containing a single DRACH motif. We manipulated these sequences to contain a 21-nucleotide perfect stem sequence with an 11-nucleotide loop, and systematically shifted the relative position of the DRACH motif from the middle of the loop (position 56) to the middle of the stem (position 72). We mutated different nucleotides further from the methylated site to generate perfect stem and loop secondary structures, in a way that the double-stranded stem structure starts from position 62 of the 101 basepairs long variable inserted segment. We designed the m6A-QTL set (Fig. 5d) by first filtering for m6A-QTLs with a *p*-value of less than 0.01 and within a 30-nt distance from the m6A enrichment window peak center [22]. We obtained 317 filtered m6A-QTLs and selected 101 m6a-QTLs, prioritized to achieve relatively balanced representation across the three groups presented in Fig. 5d. We then extracted 101-nt long sequences surrounding each of these selected m6A-QTLs from both the reference and alternative alleles.

### Supplementary Information

**Additional file 1: Figure S1.** Yeast samples methylation during meiosis progression. **Figure S2.** Cis evolution of gene methylation levels between yeast species. **Figure S3.** The relative contribution of each position around the methylated adenosine to the conservation of an m6A site in saccharomyces species. **Figure S4.** Secondary structure role in site methylation is independent of the RNAfold measured window size. **Figure S5.** Interdependency between changes in the m6A consensus motif and alterations in mRNA secondary structure **Figure S6.** m6A site scores following genetic perturbations revealing a causal role for structure. **Figure S7.** The relative frequency of DRACH motifs centered around detected m6A peak summit positions in mammalian samples. **Figure S8.** Parental versus hybrid m6A levels in the identified m6A sites (human-mouse cell lines). **Figure S9.** Trans and cisTrans m6A sites are within genes that are disproportionately lowly expressed from the human allele in the monochromosomal hybrid. **Figure S10.** Gene methylation levels between mammal species are guided by cis determinants. **Figure S11.** Classification of changes in sequence associated with the changes in methylation. **Figure S12.** m6A peak often originates from a cluster of DRAC motifs, rather than from a single site. **Figure S13.** The mice intra- species hybrid demonstrates the importance of secondary structure to allele-specific methylation. **Figure S14.** Quantitative trait loci (QTLs) maps of m6A peaks indicate the role of DRACH motif sequence in methylation differences between individuals. **Figure S15.** Divergence in m6A gene levels between humans and mice correlates negatively with allele-specific expression levels.

**Additional file 2. **The m6A-peaks detected in this study. 

**Additional file 3: **The m6A-peaks detected throughout this study in the yeast strains (**Table S3**), human-mice interspecies hybrid system (**Table S4**) and mice-mice intraspecies hybrid system (**Table S5**).

**Additional file 4. **The gene annotation files used in the saccharomyces species (**Table S6**), and the different mammals species (**Tables S7-S8**).

**Additional file 5. **Review history.

## Data Availability

The datasets generated in this study for analyzing m6A and RNA expression are deposited on Gene Expression Omnibus (GEO) under accession number GSE232450 [55]. The source code, gene annotation and data used in this study are freely available on Zenodo [56] and github under the MIT license [57]. The massively parallel reporter assay were deposited in GEO database (accession number GSE204980 [6]). m6A-QTLs data were obtained from GEO database (accession number GSE125377 [22]).
