# Supplementary material for: Dissecting the sequence and structural determinants guiding m6A deposition and evolution via inter- and intra-species hybrids
Source: Genome Biol. 2024 Feb 15;25:48. doi: 10.1186/s13059-024-03182-1 (PMC10870504; doi:10.1186/s13059-024-03182-1)
Supplement: Supplementary file 1 — Additional file 1: Figure S1. Yeast samples methylation during meiosis progression. Figure S2. Cis evolution of gene methylation levels between yeast species. Figure S3. The relative contribution of each position around the methylated adenosine to the conservation of an m6A site in saccharomyces species. Figure S4. Secondary structure role in site methylation is independent of the RNAfold measured window size. Figure S5. Interdependency between changes in the m6A consensus motif and alterations in mRNA secondary structure Figure S6. m6A site scores following genetic perturbations revealing a causal role for structure. Figure S7. The relative frequency of DRACH motifs centered around detected m6A peak summit positions in mammalian samples. Figure S8. Parental versus hybrid m6A levels in the identified m6A sites (human-mouse cell lines). Figure S9. Trans and cisTrans m6A sites are within genes that are disproportionately lowly expressed from the human allele in the monochromosomal hybrid. Figure S10. Gene methylation levels between mammal species are guided by cis determinants. Figure S11. Classification of changes in sequence associated with the changes in methylation. Figure S12. m6A peak often originates from a cluster of DRAC motifs, rather than from a single site. Figure S13. The mice intra- species hybrid demonstrates the importance of secondary structure to allele-specific methylation. Figure S14. Quantitative trait loci (QTLs) maps of m6A peaks indicate the role of DRACH motif sequence in methylation differences between individuals. Figure S15. Divergence in m6A gene levels between humans and mice correlates negatively with allele-specific expression levels. [file 13059_2024_3182_MOESM1_ESM.docx]

**Additional file 1**


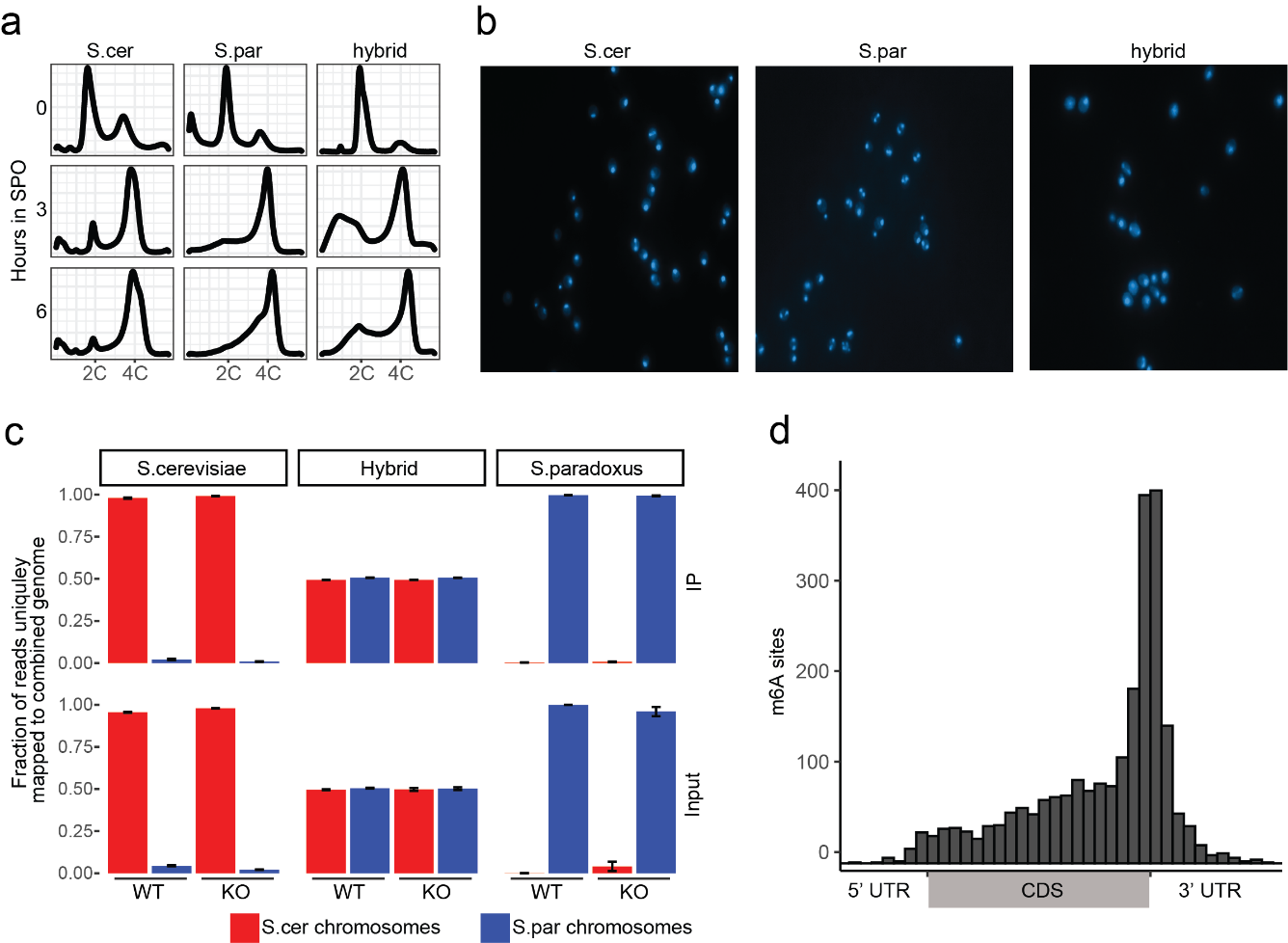


**Fig. S1 |** Yeast samples methylation during meiosis progression **(a)** Histogram showing FACS DNA quantification analysis of SYBR green at selected time points across the meiotic progression of diploid *S.cerevisiae*, *S.paradoxus*, and their mated hybrid. **(b)** Microscopy imaging of the DAPI-stained cells of the mentioned species at 4 hours after transferring the cells into the SPO medium. S.cer and the hybrid show a similar level of DNA segregation (~15%), while 50% of the S.par cells pass meiosis1 segregation at the same time point. **(c)** the fraction of reads mapped to the combined S.cer-S.par genome. Nearly 100% of each parental species’ reads are mapped to the correct genomic source, while in the hybrid strain, ~50% of the reads are mapped to each genome, both in the IP (top) and Input (bottom) **(d)** metagene distribution of the detected m6A sites.


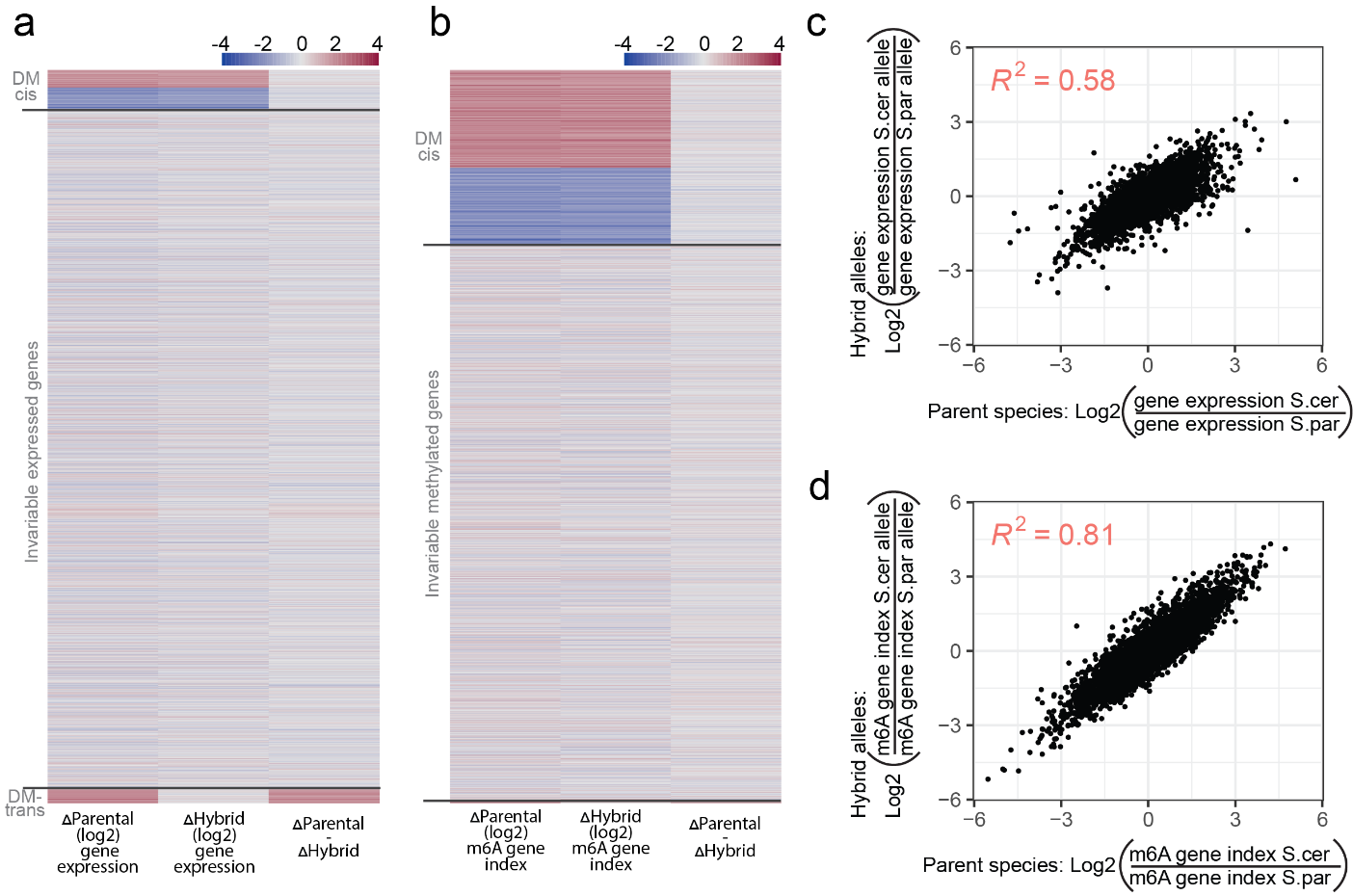


**Fig. S2 |** Cis evolution of gene methylation levels between yeast species. **(a)** clustered heatmap classification of genes with significant cis and/or trans gene expression effect above 1.4-fold or into conserved genes. The gene expression ratio between the parental strains (left column), the hybrid alleles (middle column), or the delta of both (right column). A gene labeled in red is expressed at higher levels in S. cerevisiae than in *S. paradoxus*. A gene labeled in blue is expressed at higher levels in *S. paradoxus*. Genes in various shades of white show little difference in their normalized expression between the homologs. **(b)** clustered heatmap classification of genes with significant cis and/or trans m6A gene index effect above 1.4-fold or into conserved genes. The m6A gene index ratio between the parental strains (left column), the hybrid alleles (middle column), or the delta of both (right column). A gene labeled in red is methylated at higher levels in S. cerevisiae than in *S. paradoxus*, and a gene labeled in blue is methylated at higher levels in *S. paradoxus*. Genes in various shades of white show little difference between their methylation index in the homologous alleles. **(c)** Hybrid versus parental gene expression levels in all comparable homologous genes. **(d)** Hybrid versus parental m6A gene index levels in all comparable homologous genes.


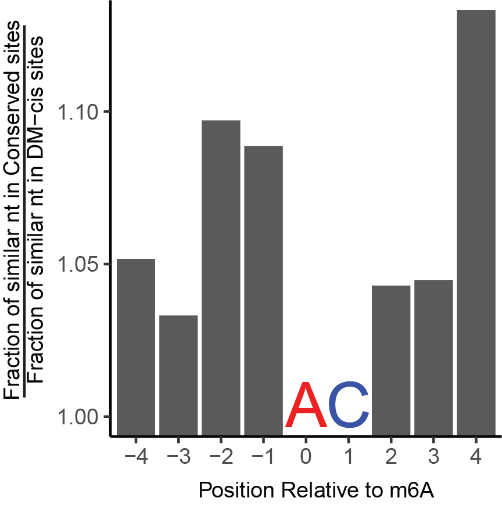


**Fig. S3 |** The relative contribution of each position around the methylated adenosine to the conservation of an m6A site. The X-axis annotates the relative position with respect to the m6A site; The Y-axis shows the normalized ratio of the position conservation in conserved sites, divided by the conservation ratio in DM-cis sites.

**
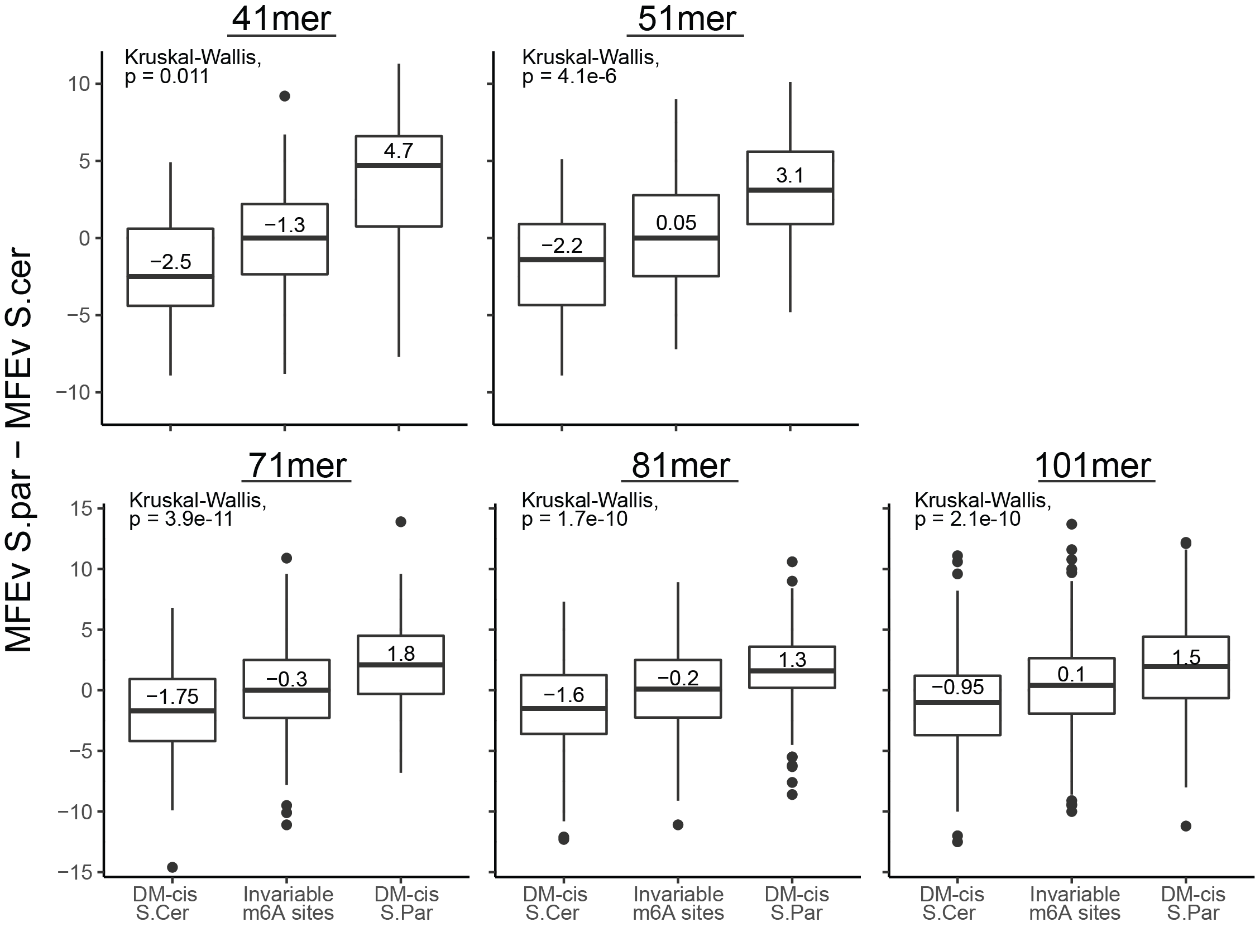
**

**Fig. S4 |** Boxplots displaying the differences between the minimum free energies values (MFE) for predicted structures on the conserved sites (both alleles are methylated, middle) and DM-cis sites (S. cerevisiae, left, *S. paradoxus*, right). The prediction is made for different sizes of windows around the detected m6A sites. Independent of the window size, sites exclusive to S. par have decreased predicted structures in S. par, and ones exclusive to S.cer have reduced predicted structures in S.cer. Sites with no variations in methylation don’t show differences in predicted structure between alleles.


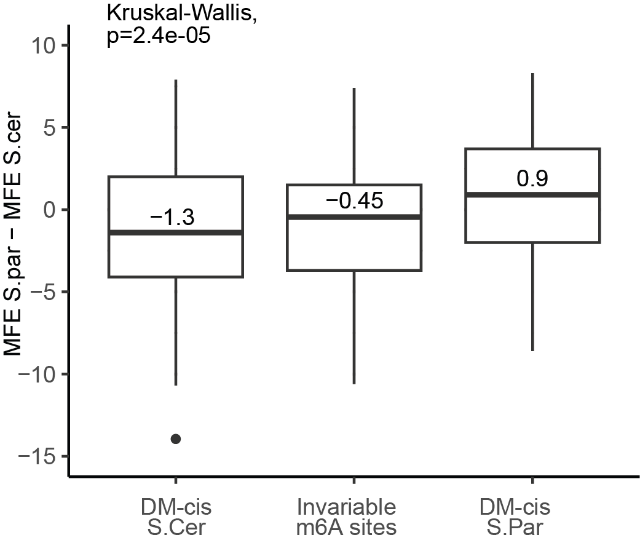


**Fig. S5 |** Boxplots displaying the differences between the minimum free energies values (MFE) for predicted structures on the Invariable m6A sites and DM-cis sites (S. cerevisiae, left, *S. paradoxus*, right). Only sites with motif-damaging mutations and at least one allele with a predicted solid structure (MFE < -8 kcal/mol) are displayed. The whiskers correspond to the value no further than 1.5× the interquartile range (n=437).

**
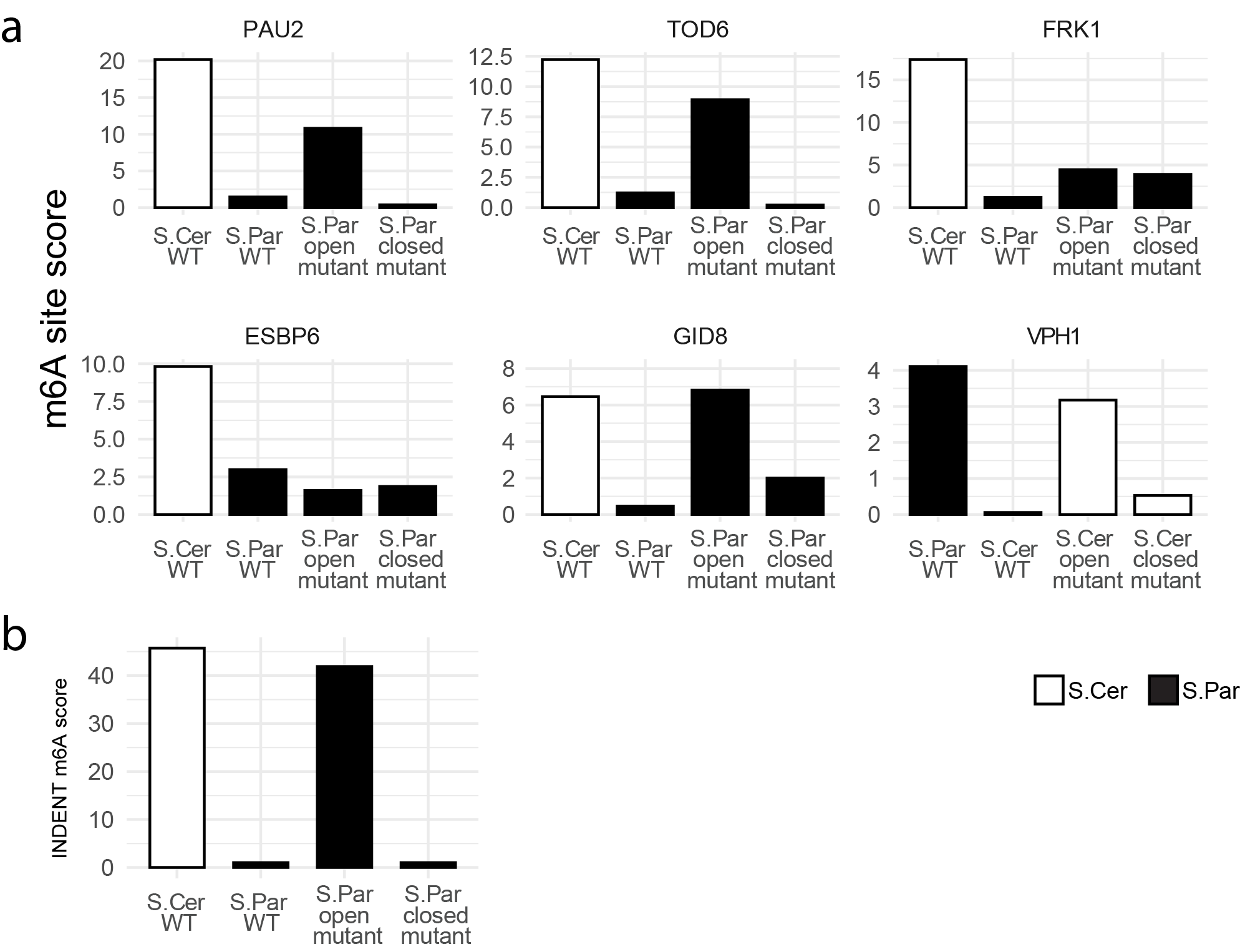
**

**Fig. S6 |** m6A site scores following genetic perturbations revealing a causal role for structure. **(a)** Measurements of m6A levels following perturbations. PAU2, TOD6, PRK1, ESBP6 and GID8 were detected as methylated exclusively in S.cer, while the m6A site in VPH1 mRNA was detected as methylated exclusively in *S.paradoxus*. Mutations predicted to first open the structure in the unmethylated allele, and then reclose the structure were designed and introduced via CRISPRs into yeast hybrids. **(b)** m6A IDENT-score for the m6A sites at PAU2. The read coverage in both IP and Input samples only considers sequencing reads that come from identical RNA fragments between the WT strain and the two mutated strains. Note that in the five remaining strains not displayed here, the designed mutations were both up- and downstream of the methylated adenosine, and hence, m6A IDENT-scores could not be calculated.

**
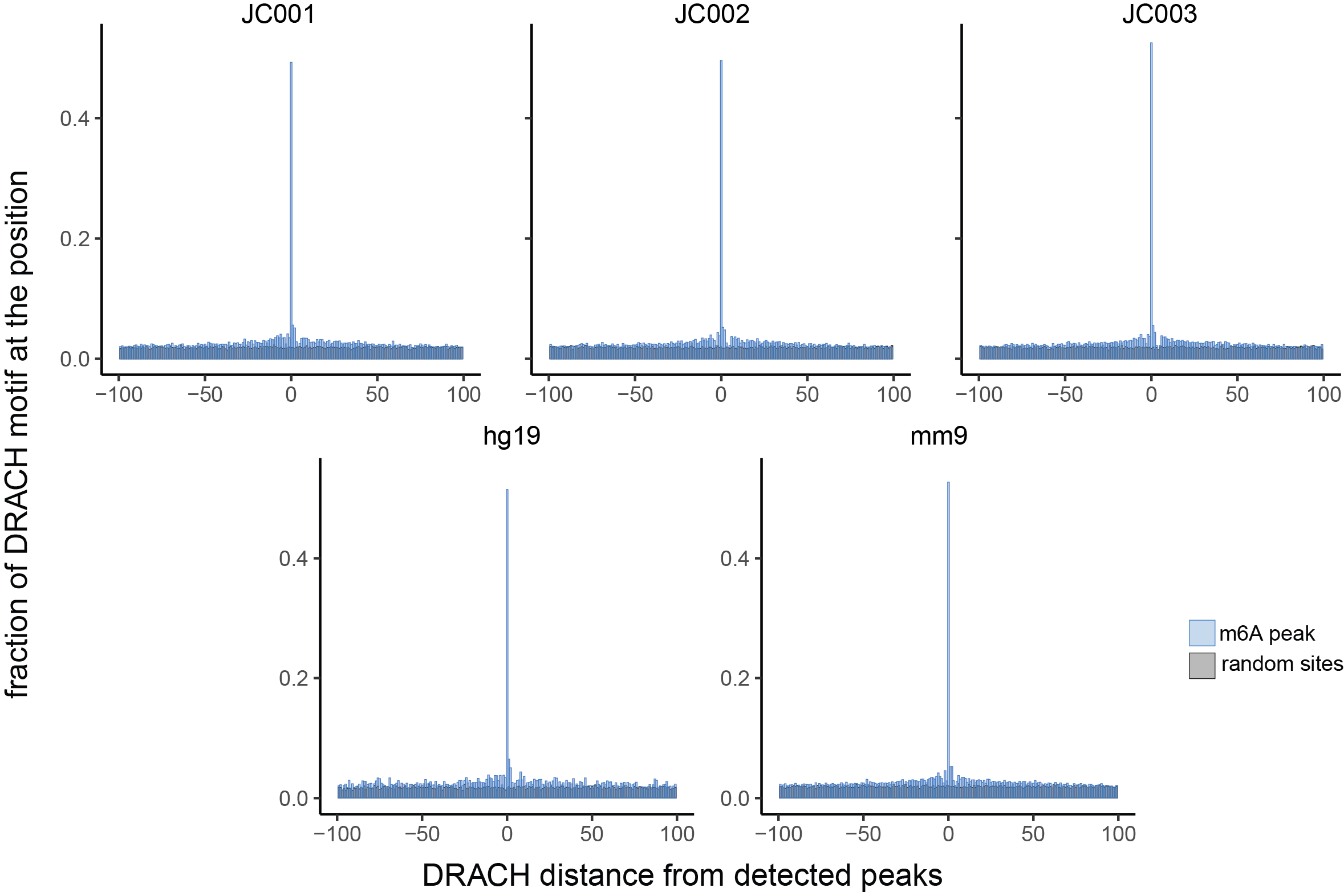
**

**Fig. S7 |** The relative frequency of DRACH motifs centered around detected m6A peak summit positions in mammalian samples (blue), in comparison to randomly sampled regions across the same genes (gray). On top, the results for the different mice cell lines containing different human source chromosomes are displayed (jc001 has human chromosome 1, jc002 has human chromosome 2, and jc003 has human chromosome 3). The lower two plots are for the parental species: human BJ cell line and mice 3T3 cell line.

**
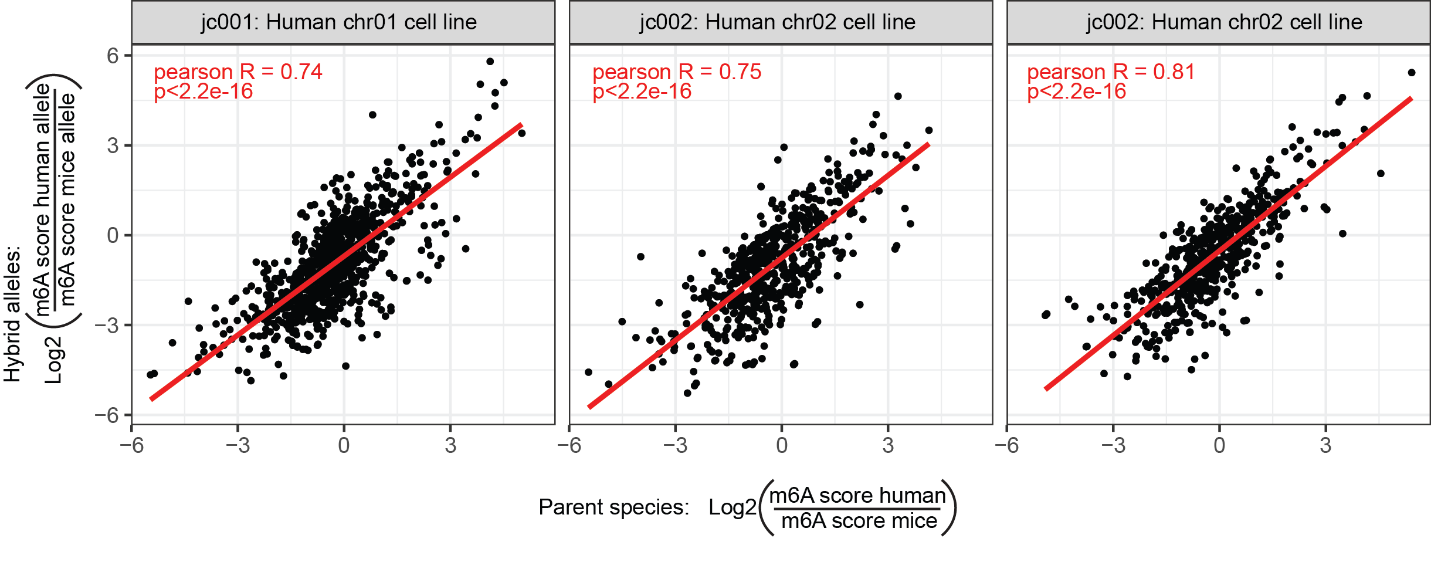
**

**Fig. S8 |** Parental versus hybrid m6A levels in the identified m6A sites. Measuring the methylation level differences between the homologous Mice-Human hybrid alleles indicate similar levels as between the parental species in all three cell lines used in this study (containing different human chromosomes).

**
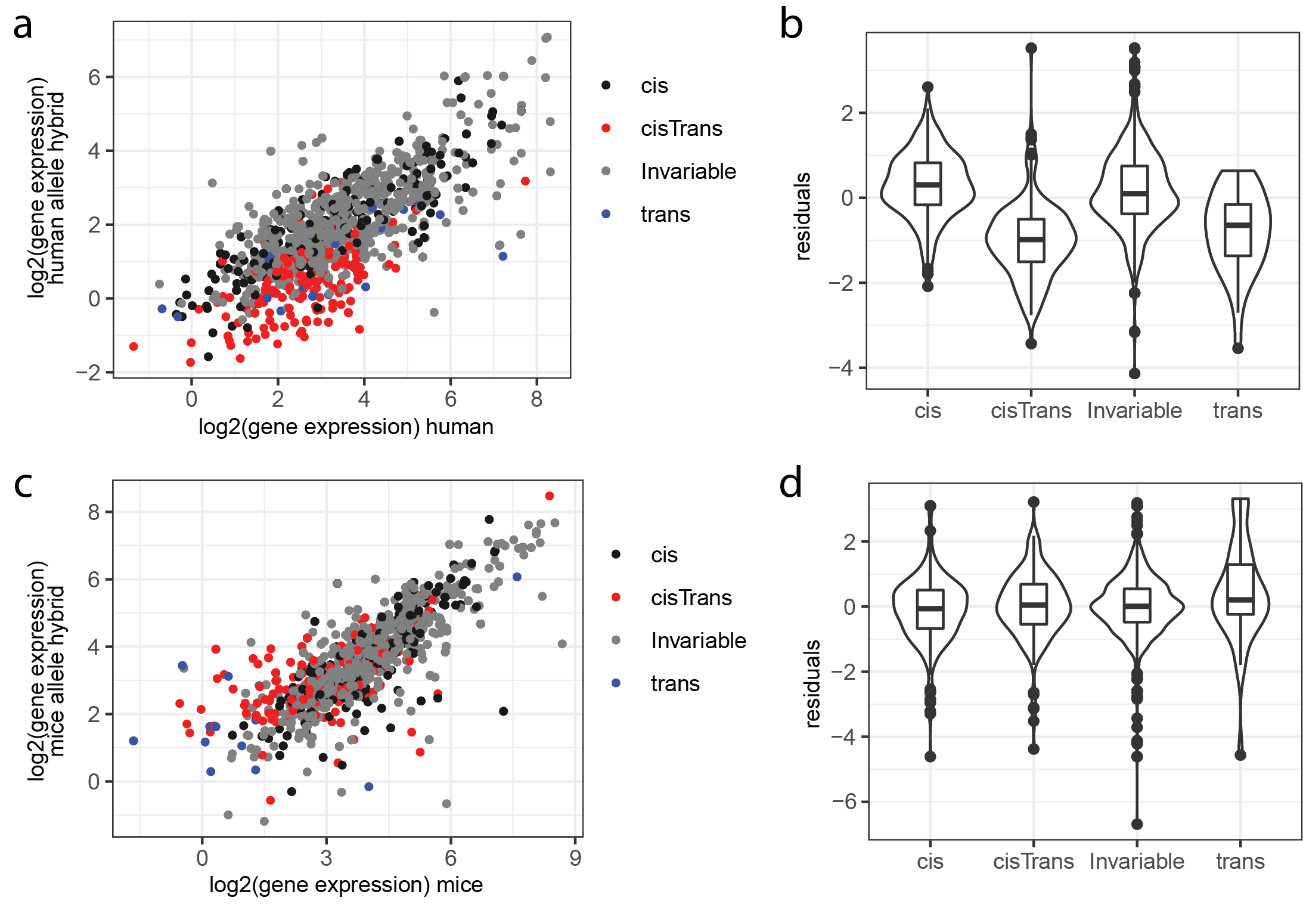
**

**Fig. S9 |** Trans and cisTrans m6A sites are within genes that are disproportionately lowly expressed from the human allele in the monochromosomal hybrid. **(a)** Scatter plots of normalized expression of each human gene (from chr1-chr3) in the BJ parent species (x axis) against its counterpart in the monochromosomal hybrid. Each gene is colored based on whether it had been classified as a ‘cis’, ‘trans’, ‘cisTrans’ or ‘Invariable’ site. Only genes with at least one detected m6A site are displayed. **(b)** violin plot depicting the distribution of residuals (calculated on the basis of (a), highlighting the increased residuals of the ‘cisTrans’ set of genes.  **(c)** Scatter plots, conducted for the genes depicted in (a) but on the basis of their mouse orthologs. X axis depicts measurements in the pseudo-parental 3T3 cells, and Y axis in the hybrid. Genes are colored based on the same classification as in (a). **(d)** violin plot, as in (b), conducted on the basis of the data depicted in (c).


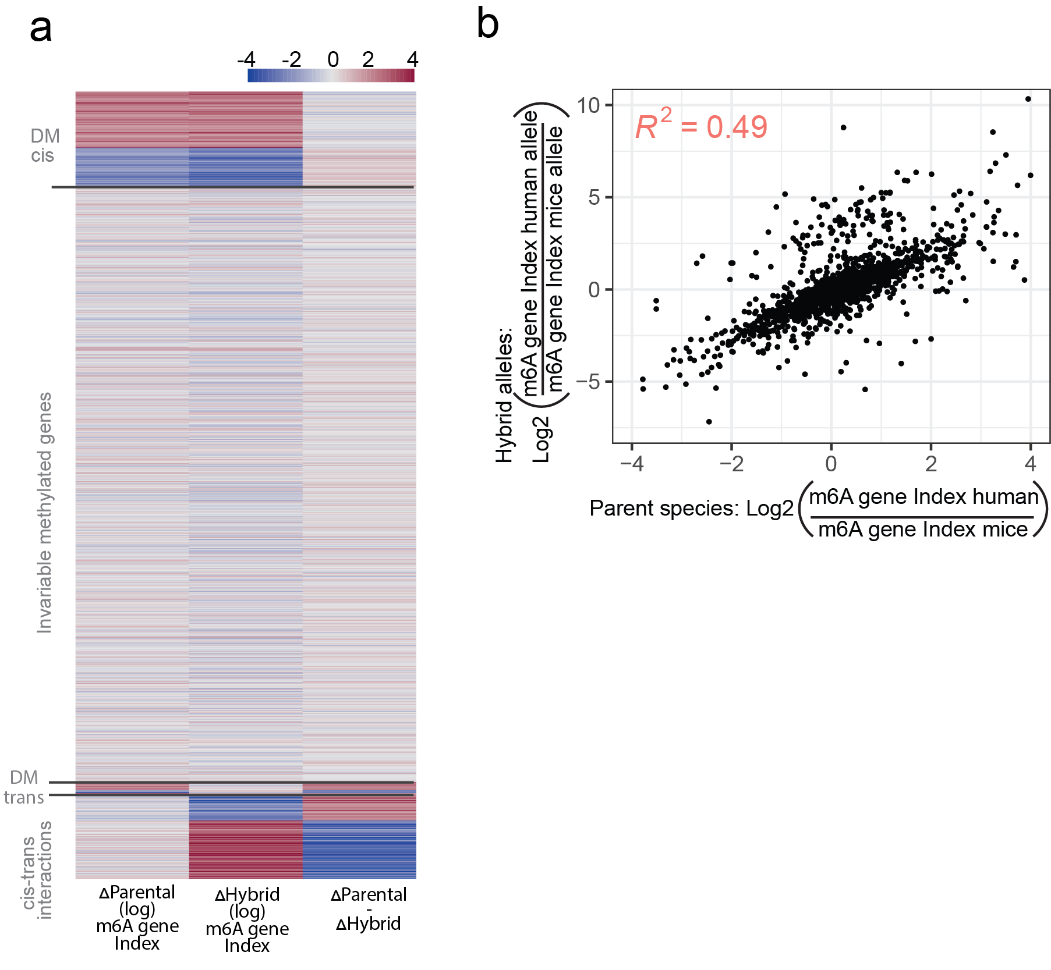


**Fig. S10 |** Gene methylation levels between mammal species are guided by cis determinants. **(a)** clustered heatmap classification of genes with significant cis and/or trans m6A gene index effect above 1.4-fold or into conserved genes. The m6A gene index log2-ratio between human and mice (left column), their corresponding alleles in the monochromosomal hybrid (middle column), or the delta of both (right column). **(b)** Parental versus hybrid m6A gene index levels in all comparable homologous genes between mice to their homologous alleles reside in human chromosomes 1, 2 or 3.

**
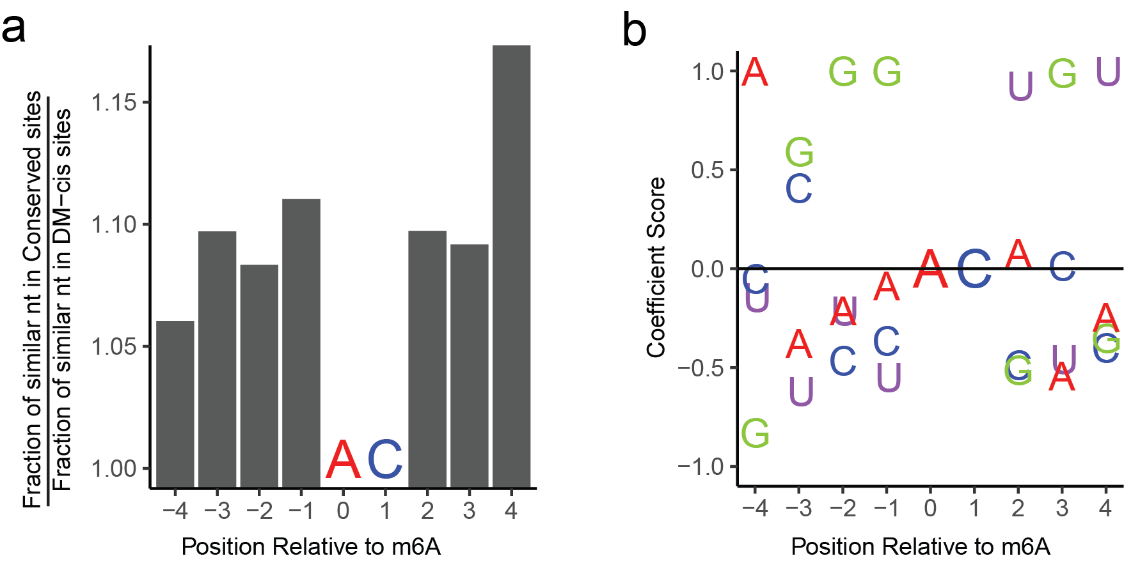
**

**Fig. S11 |** Classification of changes in sequence associated with the changes in methylation. **(a)** The relative contribution of each position around the methylated adenosine to the conservation of an m6A site in the mammal samples. The X-axis annotates the relative position with respect to the m6A site; The Y-axis shows the normalized ratio of the position conservation in conserved sites, divided by the conservation ratio in DM-cis sites. **(b)** Different nucleotides surrounding the m6A site contribute to its methylation levels in the mammal samples. The coefficient score depicts the reduction of the per-base nucleotide conservation rate in ‘differentially-methylated cis’ versus ‘invariable’ m6A sites, normalized around 0 and scaled between -1 to +1

**
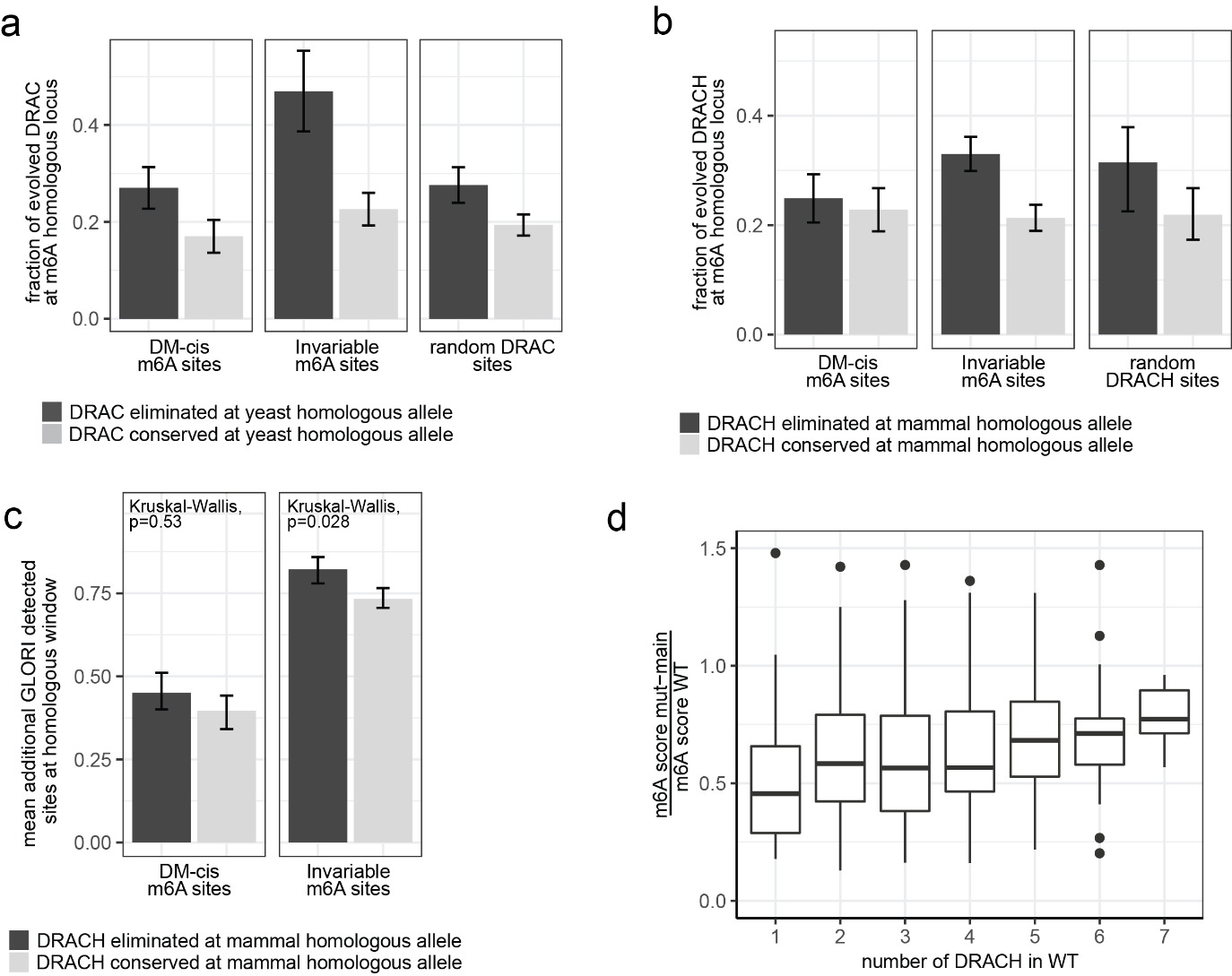
**

**Fig. S12 |** m6A peak often originates from a cluster of DRAC motifs, rather than from a single site. **(a)** Analysis of the fraction of sites in which an additional DRAC motif is present within a 61-nt window from the detected m6A motif, separately for sites in which the DRAC motif was conserved or not-conserved across both alleles and binned separately for ‘cis’, ‘trans’ and a randomly selected set of sites. This analysis highlights that in the subset of sites that are considered ‘invariable’ and yet in which the DRAC motif was eliminated in one of the alleles, there is a strong enrichment in terms of adjacent DRAC motifs in the motif-disrupted allele. **(b)** similar analysis as in (a), displayed for sites in mammals. **(c)** The number of additional m6A sites detected in the GLORI dataset around the DM-cis and Invariable m6A sites , revealing an enrichment for methylated residues around sites clustered as ‘invariable’, independently of whether or not the motif was maintained. **(d)** Boxplot depicting the contribution of the auxiliary DRACH motif to the total measured m6A signal, based on 101-nt long sequences surrounding 400 methylated sites from human and mice [^6^](https://paperpile.com/c/f6sGM8/oT1F). The Y-axis shows the normalized m6A enrichment values (IP/input) ratio for the sequences with point mutated main motif versus WT, revealing that an increasing number of proximal DRACH motifs allows compensating for loss of the main motif. Boxplots correspond to the median, Q1 and Q3, whiskers mark Q1-1.5 IQR and Q3+1.5 IQR.


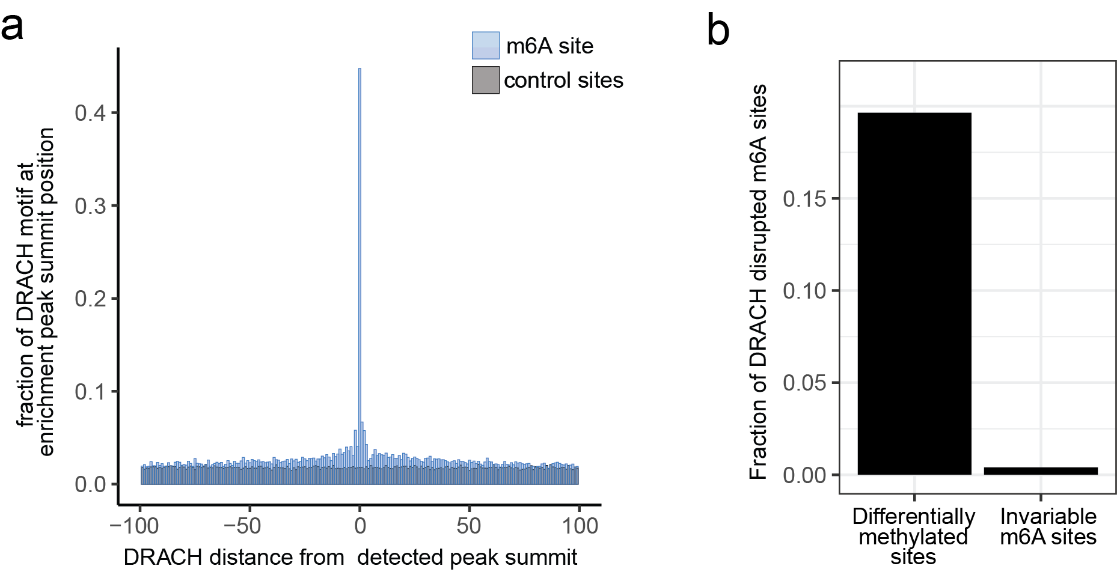


**Fig. S13 |** The mice intra- species hybrid demonstrates the importance of secondary structure to allele-specific methylation. **(a)** The distribution of distances between the peak summit positions identified in this study and the transcriptomic close-by mice m6A consensus DRACH motifs (green) compared to randomly sampled regions across the same genes (gray). **(b)** Bar plot showing the fraction of m6A sites with disrupted DRACH motif within differentially methylated sites (left) or invariable ones (right).


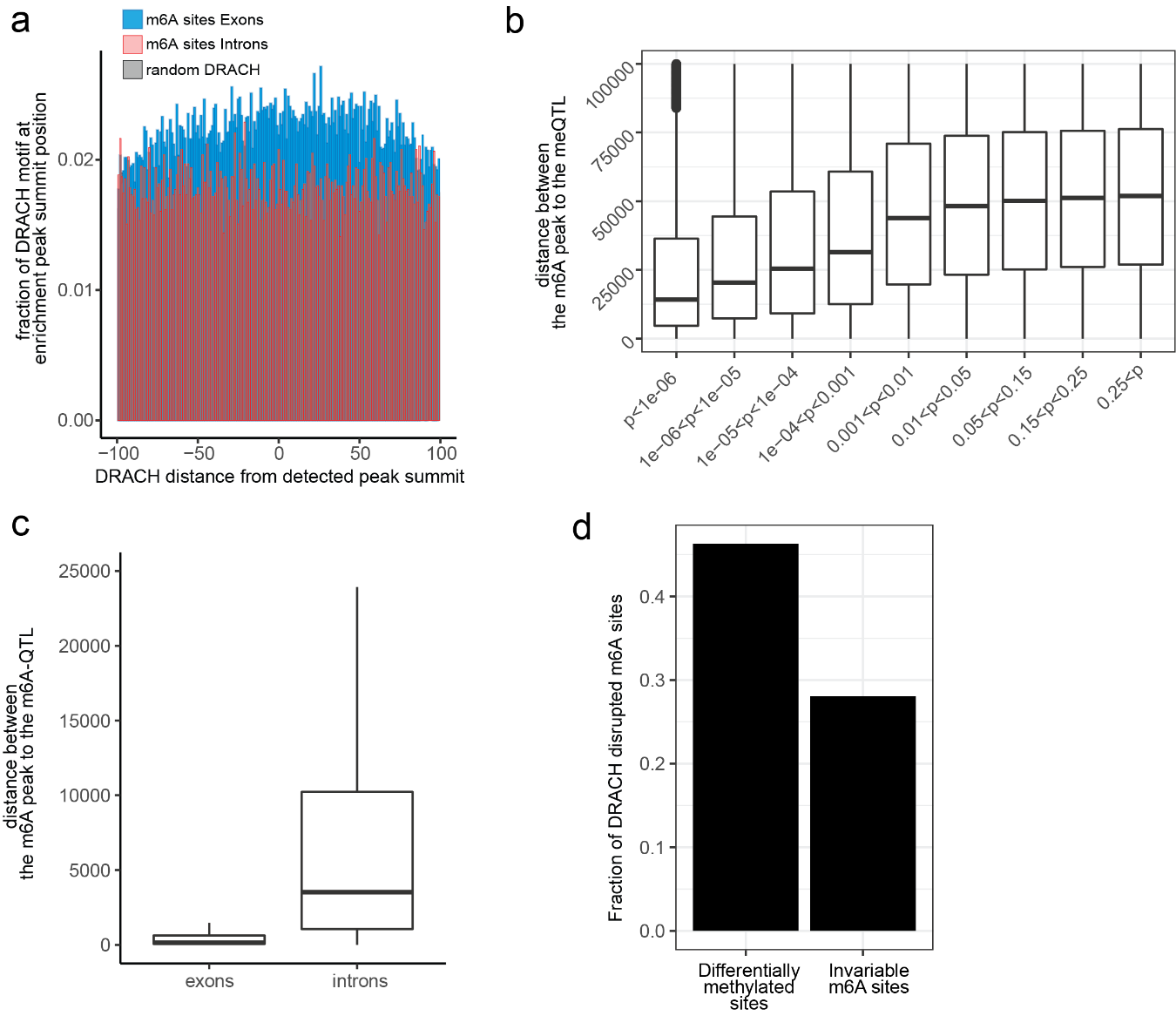


**Fig. S14 |** Quantitative trait loci (QTLs) maps of m6A peaks study (Zhang et al.) indicate the role of DRACH motif sequence in methylation differences between individuals. **(a)** The distribution of distances between the peak summit positions identified in Zhang et al. (pVal<0.01) and the nearest DRACH motif in transcriptional space for exon-detected peaks (blue), introns-detected peaks (red), and randomly sampled nucleotides across the same genes (gray). Intron-detected peaks exhibit a similar ~0.175 random chance of a DRACH motif across the examined region, while the exon-detected peaks show increased proximity to DRACH motifs. **(b)** Distribution of distances between m6A peaks and meQTL (Y axis), binned as a function of P value of the association between the two (X axis). Boxplots correspond to the median, Q1 and Q3; whiskers mark Q1-1.5 IQR and Q3+1.5 IQR. **(c)** Boxplot showing the differences between exon-located QTLs to intron-located QTLs in terms of the distance of the m6A peak to the m6A-QTL. **(d)** Bar plot showing the fraction of m6A sites with conserved vs. eliminated DRACH motif between the reference to alternative alleles.

**
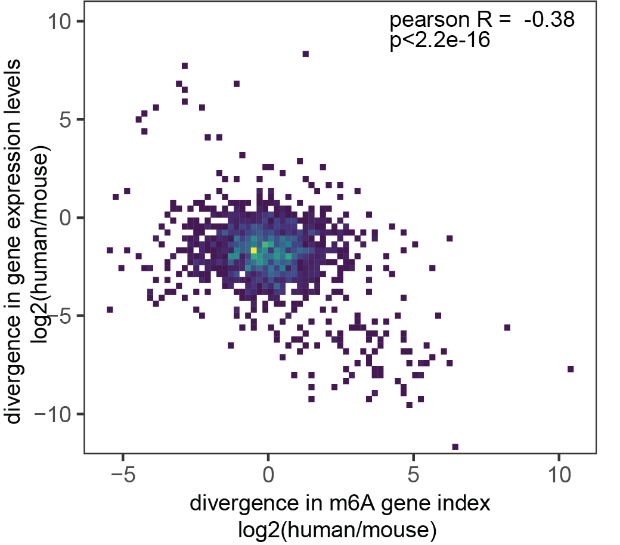
**

**Fig. S15 |** Divergence in m6A gene levels between humans and mice correlates negatively with allele-specific expression levels. Results displayed are for human genes located at chromosomes 1, 2, or 3 and homologous alleles in mice, on the basis of measurements in the monochromosomal cell line. Divergence in gene expression is portrayed as the log fold-change between human and mouse gene expression, while variation in m6A levels is computed as the log fold-change between human and mouse m6A gene index.
