# Supplementary material for: Dissecting the sequence and structural determinants guiding m6A deposition and evolution via inter- and intra-species hybrids
Source: Genome Biol. 2024 Feb 15;25:48. doi: 10.1186/s13059-024-03182-1 (PMC10870504; doi:10.1186/s13059-024-03182-1)
Supplement: Supplementary file 5 — Additional file 5. Review history. [file 13059_2024_3182_MOESM5_ESM.docx]

Review History

**First round of review**

**Reviewer 1**

**Were you able to assess all statistics in the manuscript, including the appropriateness of statistical tests used?**

Yes, and I have assessed the statistics in my report.

**Comments to author:**

In this study, the authors utilized a hybrid of two yeast species to investigate the genetic architecture of a type of RNA modification, N6-methyladenosine (m6A). By comparing methylation levels in two parental strains and allele-specific methylation levels in the hybrid, the authors tested whether methylation level divergence between two parental species is due to cis- or trans- divergence, and found the former is much more prevalent. Upon closer examination of the nature of substitutions associated with methylation divergence, the authors identified two molecular mechanisms of methylation level change: change in the consensus motif, and alteration of mRNA secondary structure. Using a similar approach, the authors also examined m6A divergence between human and mouse, and similarly found cis- divergence to be the main mechanism underlying methylation level differences. In addition, the authors found gene-level m6A divergence is associated with divergence in expression levels, revealing a downstream consequence of m6A evolution.

Overall, this study is well designed, and the manuscript is mostly clearly written, though I have some relatively minor questions and comments.

Note that I will use "line X, page Y" to refer to locations in the manuscript, as line numbers are restarted every page. Page number is based on the PDF file I downloaded from the journal's system. As line numbers are often not well aligned with the text, I may use a broader range of lines that include the text I refer to (e.g., line 10-11 if the line I refer to lies between line 10 and 11).

Comment 1

The authors made a distinction between two mechanisms of methylation level divergence: change in the consensus motif, and change in mRNA secondary structure.

However, as the authors only predicted and analyzed the secondary structure for a subset of m6A sites with intact consensus motifs, it is unclear to what extent the two mechanisms are independent - i.e., substitution in the motif may also cause change in mRNA secondary structure. If motif changes generally cause secondary structure changes as well, it seems that the two mechanisms are not really distinct but essentially the same one.

Thus, I wonder in how many cases methylation divergence can be attributed exclusively to one mechanism, and in how many cases the two mechanisms' effects are indistinguishable.

Comment 2

Although this study is about the evolution of m6A levels, a key question remains unanswered: is the observed divergence (of lack of divergence) mainly driven by selection, or genetic drift? While I understand it may be beyond the scope of this study to systematically address this question, more discussion about it is desired.

Specifically, the author mentioned that there have been compensatory evolutionary changes that maintained gene-level methylation, and I think it is worth discussing the cause of compensatory evolution - e.g., is it due to stabilizing selection on the overall methylation level of a gene and/or its expression level? The authors are also encouraged to discuss this in light of other types of compensatory evolution, such as that between transcription and translation driven by stabilizing selection on the protein level.

Minor comments

Line 11, page 7: It is more accurate to use "substitution" instead of "mutation".

Line 51-52, page 10, and Fig. 2 legend: "SNP" is used to refer to interspecific difference here. It should be "substitution".

Line 21, page 11: Though quotation marks are used, the word "neutral" still feels confusing. It may be better to replace "'neutral' change" with "expectation".

Line 53-55, page 13: What are the criteria for the choice? It is desirable to have them briefly described in the Results section.

**Reviewer 2**

**Were you able to assess all statistics in the manuscript, including the appropriateness of statistical tests used?**

Yes, and I have assessed the statistics in my report.

**Comments to author:**

In this work, Shachar et al aim to explore the evolution of m6A-mediated gene regulation, and identify the factors that drive differences in where m6A is deposited in different species. In particular, they use interspecies hybrids (yeast and human/mouse) to more precisely dissect the impacts of cis and trans factors. Both analyses appear to support the conclusion that changes in m6A across species are derived from changes in sequence (or cis factors). The authors take it a step further to determine if/how local structure may impact m6A-deposition changes that are not driven by mutations within the consensus motif, and support these analysis with specific perturbations to the sequences of interest that perturb or restore structure in those regions. Overall the approach of using interspecies hybrids to more rigorously dissect contributions of cis and trans factors to m6A deposition is conceptually powerful, but there are some weaknesses in the implementation and analysis (some of which the authors acknowledge in the text). The authors have also curiously not cited or compared their results to other studies that have tried to address these questions (details below). As such, at minimum this work would require written revisions in order to be appropriate for publication.

1. Other studies have sought to address the question of what factors drive changes in m6A deposition across different species, including Ma et al, "Evolution of transcript modification by N6-methyladenosine in primates", Genome Research, 2017. Though this study also arrived at similar conclusions with respect to the fact that changes in the consensus sequence are a significant driver of m6A deposition, this study is not mentioned or cited. The authors should include a detailed discussion of how their work fits in with this and other studies, including whether their conclusions agree or disagree, and how/whether experimental or analytical differences influence these conclusions.

2. While the authors recognize the need for high resolution data to perform these analyses, they have used a method that is inherently not high resolution. While the changes implemented in m6A-seq2 are commendable and certainly improve the quality of the data, the 'stringent computational filtering' imposed is not sufficient to turn the data into, effectively, single nucleotide resolution data. Peaks can be quite wide, and the summit is apparently used to center the peak, with the argument that the median distance to a consensus site is 1nt. However, the authors note that random sampling yields a median distance of 14 nt, which on the scale of the widths of these peaks still seems a bit concerning. The authors themselves note later in the manuscript that multiple consensus sequences in clusters may introduce robustness into m6A-mediate regulation, and yet do not seem consider how such clusters might affect 'assigning' sites to specific consensus sequences in these analyses. Related to this point, the authors state that "in 70% of differentially methylated sites unique to S. paradoxus, the methylation motif was disrupted in S. cerevisiae. Conversely, among the sites with conserved m6A levels between the two species, a motif disruption was present in only 32% of cases". The authors point to structure accounting for at least part of this 32%, but a possible interpretation that is not mentioned is that there is m6A signal being assigned to an area with motif disruption that may be the result of mis-assignment of some sites or that the cut off for enrichment was too stringent and there is actually signal nearby that is not 'detected' in the analysis. While the role of structure is interesting in and of itself, there may be limitations in the analysis that offer alternative explanations.

3. The analysis correlating with m6A-mediated decay near the end of the manuscript is weak and should either be done more thoroughly or removed. To make a claim about m6A-mediated decay mechanisms, one should really be looking at the half-life of different transcripts, not just gene expression levels which will also be influenced by transcription and other processing steps.

4. A more minor point that might be worth addressing is whether the fact that m6A methylation in yeast is restricted to meiosis may impact how conserved sites are across yeast. This is in contrast to the mammalian setting, where m6A methylation (particularly m6A-mediated mRNA decay) is a more broadly utilized regulatory mechanism across many different biological processes. That being said, this reviewer studies RNA modifications and is not an evolutionary biologist, so I would defer to experts in that area.

**Authors Response**

**Point-by-point responses to the reviewers’ comments:**

Reviewer #1:

In this study, the authors utilized a hybrid of two yeast species to investigate the genetic architecture of a type of RNA modification, N6-methyladenosine (m6A). By comparing methylation levels in two parental strains and allele-specific methylation levels in the hybrid, the authors tested whether methylation level divergence between two parental species is due to cis- or trans- divergence, and found the former is much more prevalent. Upon closer examination of the nature of substitutions associated with methylation divergence, the authors identified two molecular mechanisms of methylation level change: change in the consensus motif, and alteration of mRNA secondary structure. Using a similar approach, the authors also examined m6A divergence between human and mouse, and similarly found cis- divergence to be the main mechanism underlying methylation level differences. In addition, the authors found gene-level m6A divergence is associated with divergence in expression levels, revealing a downstream consequence of m6A evolution.

Overall, this study is well designed, and the manuscript is mostly clearly written, though I have some relatively minor questions and comments.

Note that I will use "line X, page Y" to refer to locations in the manuscript, as line numbers are restarted every page. Page number is based on the PDF file I downloaded from the journal's system. As line numbers are often not well aligned with the text, I may use a broader range of lines that include the text I refer to (e.g., line 10-11 if the line I refer to lies between line 10 and 11).

Comment 1

The authors made a distinction between two mechanisms of methylation level divergence: change in the consensus motif, and change in mRNA secondary structure.

However, as the authors only predicted and analyzed the secondary structure for a subset of m6A sites with intact consensus motifs, it is unclear to what extent the two mechanisms are independent - i.e., substitution in the motif may also cause change in mRNA secondary structure. If motif changes generally cause secondary structure changes as well, it seems that the two mechanisms are not really distinct but essentially the same one.

Thus, I wonder in how many cases methylation divergence can be attributed exclusively to one mechanism, and in how many cases the two mechanisms' effects are indistinguishable.

*Reply: We appreciate the insightful observation. We completely agree with the intuition of this reviewer that sequence and structure are often indistinguishable, as it is impossible to modify the sequence without altering the structure, at least to some extent. Nonetheless, we do choose to discriminate between the two, given that the mechanism via which each of these impacts methylation is likely distinct. The requirement for the consensus motif likely reflects critical molecular contacts between the RNA methylation machinery and the target RNA that take place during enzymatic m6A catalysis. In contrast, the local secondary structure of a target likely impacts methylation by restricting the accessibility of the megadalton methylation machinery to a target site, in a manner that likely does not depend on individual contacts.*

*In view of the reviewer’s comment, we have now investigated the potential interdependence between changes in the m6A consensus motif and alterations in mRNA secondary structure. To test this relationship, we now explore predicted secondary structures at sites in which the methylation consensus motif is not intact (Reviewer Figure 1, right panel). Interestingly - and in line with the hypothesis raised by this Reviewer - our analysis reveals that differences in secondary structure persist even within this subset of sites. Importantly, the differences in secondary structures between the three groups (cis-Cer, invariable, cis-Par) are substantially lower than within the set of sites with intact m6A consensus sites (Reviewer Figure 1, left panel), consistent with the notion that the changes are primarily driven by sequence than by structure, and yet the fact that the structural differences persist indicate that structure likely impacts this group as well. We have incorporated these additional analyses into our revised manuscript, including a new supplementary figure (new Supplementary Figure S4), and a section in the Results.*

*Revision Figure 1. Boxplot illustrating minimum free energy variations (MFE) for predicted RNA secondary structures between S. cerevisiae (S. cer) and S. paradoxus (S. par). The plot is divided into two categories: 'Intact motif' and 'Disrupted motif.' Within each category, data is further classified into 'DM-cis S. cer' (m6A sites exclusively in S. cer), 'DM-cis S. par' (m6A sites exclusively in S. par), and 'Invariable' (sites with consistent methylation levels in both alleles). Predictions cover a 61-nt window centered around the methylated adenosine. Only sites with a predicted stable structure (MFE < -8 kcal/mol) in at least one of the homologous alleles are included. Whiskers extend no further than 1.5 times the interquartile range (n=360 for 'Intact motif,' n=437 for 'Disrupted motif').*

Comment 2

Although this study is about the evolution of m6A levels, a key question remains unanswered: is the observed divergence (of lack of divergence) mainly driven by selection, or genetic drift? While I understand it may be beyond the scope of this study to systematically address this question, more discussion about it is desired.

Specifically, the author mentioned that there have been compensatory evolutionary changes that maintained gene-level methylation, and I think it is worth discussing the cause of compensatory evolution - e.g., is it due to stabilizing selection on the overall methylation level of a gene and/or its expression level? The authors are also encouraged to discuss this in light of other types of compensatory evolution, such as that between transcription and translation driven by stabilizing selection on the protein level.

*Reply: Indeed, this is a cardinal point. Unfortunately, our work was neither designed to address this point, nor (with sampling of only two species) is it powered to do. We have now enhanced our discussion section, to better place our findings in the perspective of some previous work conducted on these questions in the past. We also better clarify our thoughts on the possibility of stabilizing selection being applied at the gene level - rather than at the site level - to maintain a fixed gene expression level. We do not believe that the types of measurements we have obtained allow us to make definitive inferences regarding evolutionary forces. We now highlight that while we see some evidence for reduced heterogeneity at the gene level, than at the site level,, we cannot rule out that the more conserved patterns at the gene level, than at the site level, are in part also due to the fact that variability of the mean (in this case: m6A-GI over entire genes, reflecting signal originating from multiple methylation events) is lower than variability of individuals (in this case: individual sites). For example, under a scenario wherein in each species DRAC motifs are selected for methylation randomly at a fixed propensity (e.g. 5% of DRAC motifs are methylated), substantial fluctuations between two alleles would be evident at the level of individual sites, but such changes would appear buffered at the gene level.*

*We now discuss these points in the Discussion section.*

Minor comments

Line 11, page 7: It is more accurate to use "substitution" instead of "mutation".

*Reply: Corrected.*

Line 51-52, page 10, and Fig. 2 legend: "SNP" is used to refer to interspecific difference here. It should be "substitution".

*Reply: We have corrected all of the instances in which SNP was used.*

Line 21, page 11: Though quotation marks are used, the word "neutral" still feels confusing. It may be better to replace "'neutral' change" with "expectation".

*Reply: The sentence was modified to ‘These frequencies were normalized by their counterparts in the m6A conserved set of sites, with the rationale that these should reflect a baseline of changes that do not impact m6A formation’.*

Line 53-55, page 13: What are the criteria for the choice? It is desirable to have them briefly described in the Results section.

*Reply: We are grateful for this opportunity to clarify our criteria. The criteria we had employed were relatively loosely defined, and included the following considerations: (1) We prioritized sites with clear-cut differences between the two species, (2) We prioritized m6A sites that lacked additional m6A peaks in close proximity, in both species, to avoid leakage of enrichment signal from one site to another, (3) We prioritized sites predicted to harbor a well-defined and interpretable structure (e.g. a stem loop), (4) We required that in this predicted structure, the methylated adenosine and its immediate neighbors be paired with other nucleotides in the unmodified allele, and (5) Finally, we prioritized sites in which minimal genetic intervention (via nucleotide substitutions) could lead to a maximal alteration of the predicted secondary structure. We have now updated the Methods section of the manuscript to clarify these criteria.*

**Reviewer 2**

**Were you able to assess all statistics in the manuscript, including the appropriateness of statistical tests used?**

Yes, and I have assessed the statistics in my report.

**Comments to author:**

In this work, Shachar et al aim to explore the evolution of m6A-mediated gene regulation, and identify the factors that drive differences in where m6A is deposited in different species. In particular, they use interspecies hybrids (yeast and human/mouse) to more precisely dissect the impacts of cis and trans factors. Both analyses appear to support the conclusion that changes in m6A across species are derived from changes in sequence (or cis factors). The authors take it a step further to determine if/how local structure may impact m6A-deposition changes that are not driven by mutations within the consensus motif, and support these analysis with specific perturbations to the sequences of interest that perturb or restore structure in those regions. Overall the approach of using interspecies hybrids to more rigorously dissect contributions of cis and trans factors to m6A deposition is conceptually powerful, but there are some weaknesses in the implementation and analysis (some of which the authors acknowledge in the text). The authors have also curiously not cited or compared their results to other studies that have tried to address these questions (details below). As such, at minimum this work would require written revisions in order to be appropriate for publication.

1. Other studies have sought to address the question of what factors drive changes in m6A deposition across different species, including Ma et al, "Evolution of transcript modification by N6-methyladenosine in primates", Genome Research, 2017. Though this study also arrived at similar conclusions with respect to the fact that changes in the consensus sequence are a significant driver of m6A deposition, this study is not mentioned or cited. The authors should include a detailed discussion of how their work fits in with this and other studies, including whether their conclusions agree or disagree, and how/whether experimental or analytical differences influence these conclusions.

*Reply: We are grateful to this reviewer for directing us to this reference. While we had already included in our manuscript two references on this topic (Xiong et al. (21) and Liu et al. (42)) we had indeed omitted this reference. We would like to highlight two fundamental gaps, both in approach and hence also in findings, between our study and this study. First, when m6A maps are compared across species, as had been conducted in this study, it is formally impossible to conclude whether observed changes are due to changes ‘in cis’ as opposed to ‘in trans’. Our study, involving a comparison of hybrid species to their parental strains, provides a formal framework via which to overcome this limitation. Second, in our study we went to long lengths to calibrate the m6A measurement approach, to obtain both very high (nearly single nucleotide) resolution and in a manner that is applied in a multiplexed manner to different samples (parental and hybrids) allowing quantitative comparison across different samples. Such measurements had not been available in the Ma et al study. In the absence of these two layers, the above study is limited in its ability to draw mechanistic inferences with respect to the forces underlying changes in methylation at individual sites. Consequently, the two studies diverge substantially in their focus: the Ma et al study focuses primarily on quantifying the evolutionary selection forces acting on methylation consensus sequences, finding some evidence for selection. Our study focuses more on the mechanistic forces, shaping RNA methylation landscapes, unravels the cis-based evolution of m6A, and uncovers the global role played by mRNA secondary structure.*

2. While the authors recognize the need for high resolution data to perform these analyses, they have used a method that is inherently not high resolution. While the changes implemented in m6A-seq2 are commendable and certainly improve the quality of the data, the 'stringent computational filtering' imposed is not sufficient to turn the data into, effectively, single nucleotide resolution data. Peaks can be quite wide, and the summit is apparently used to center the peak, with the argument that the median distance to a consensus site is 1nt. However, the authors note that random sampling yields a median distance of 14 nt, which on the scale of the widths of these peaks still seems a bit concerning.

*Reply: First, as we also highlight in the ‘limitations’ section of the manuscript, we are in complete agreement that, under an ideal scenario, this work would have been conducted with a single-nucleotide approach for monitoring m6A levels. Unfortunately, when we embarked on this project over 4 years ago, methodologies allowing robust, high-complexity and unbiased m6A monitoring at single nucleotide resolution did not exist. Of note, to obtain interpretable maps in hybrids, it is critical to have access to relatively long reads to allow unambiguous mapping to the genome. Methods such as m6A-miCLIP or MAZTER-seq (which did exist at the time) both suffered from limitations of complexity and read lengths, which would have precluded these analyses. At the time we therefore opted to optimize m6A-seq2 to the best of our abilities, which we were able to achieve to a considerable extent, and to reach resolutions that are very near to those obtained in approaches that are formally considered “single-nucleotide”. We will further highlight that even today, nearly a year following the publication of two single-nucleotide resolution methodologies for monitoring m6A 1,2, these have, to the best of our knowledge, not been adopted yet by any other group for routine monitoring of m6A, highlighting both the need for extensive knowhow, the need for protocol optimization, and the limited availability of reagents required for these protocols. Our group has been actively trying to adopt these approaches in our lab, but they do not yet work in a sufficiently robust manner to allow them to be applied to the questions that we have dissected in our study. Moreover, given that both these approaches rely on converting all modified adenosines (but not m6A) to guanosines thereby reducing the information content of sequencing reads, these approaches are poorly suited to being applied to hybrid species, due to challenges in uniquely aligning them to the proper allele of origin.*

*Finally, we would like to address the concern pertaining to the 14 nt distance of random peaks, and perhaps put these numbers into perspective by providing some additional statistics. By chance, when randomly sampling peaks, they occur at a median distance of 14 nt from the nearest DRAC motif, with only 7% of peaks occurring within 1 nt of a DRAC motif. In contrast, in our optimized version of m6A-seqs2, 57% of peaks occur within 1 nt of a DRAC motif. This establishes that the overwhelming majority of sites detected within a 1 nt window from a DRAC motif are likely bona-fide m6A sites, especially considering that nearly no peaks whatsoever were identified when flipping the identities of the WT and KO samples. Nonetheless, we do agree with the reviewer that given the lack of formal single nucleotide resolution, putative sites detected at increasing distances from consensus motifs may reflect, in part, misassigned sites.*

2. The authors themselves note later in the manuscript that multiple consensus sequences in clusters may introduce robustness into m6A-mediate regulation, and yet do not seem consider how such clusters might affect 'assigning' sites to specific consensus sequences in these analyses. Related to this point, the authors state that "in 70% of differentially methylated sites unique to S. paradoxus, the methylation motif was disrupted in S. cerevisiae. Conversely, among the sites with conserved m6A levels between the two species, a motif disruption was present in only 32% of cases". The authors point to structure accounting for at least part of this 32%, but a possible interpretation that is not mentioned is that there is m6A signal being assigned to an area with motif disruption that may be the result of mis-assignment of some sites or that the cut off for enrichment was too stringent and there is actually signal nearby that is not 'detected' in the analysis. While the role of structure is interesting in and of itself, there may be limitations in the analysis that offer alternative explanations.

*Reply: We appreciate the reviewer's attention and acknowledge that our initial submission may have caused some confusion. Our interpretation of the data is fully consistent with that of the reviewer. Indeed, we noted that in 32% of the cases in which conserved m6A enrichment was observed in both species, the motif - at the site assigned as the methylation site - was disrupted. For this subset of sites, we do not believe structure plays any role (we invoked structure as playing a role primarily among the sites in which m6A was disrupted between two species), and apologize if our text, which we have meanwhile updated, had been unclear on this. Instead, as illustrated in Figure S11, we indeed observed a higher fraction of additional DRAC motifs, which we term ‘evolved DRAC’, in proximity to the homologous loci, which is indeed consistent with the notion that in this subset of sites the persistence of m6A enrichment signal, at both loci, is due to the appearance of DRAC motif in close proximity to the originally detected m6A site. We recognize, and also acknowledge in the manuscript, that the lack of full single nucleotide resolution of our measurements can lead to such signal leakage, as is manifested in these cases. We have revisited the Results and the Discussions sections of the manuscript to better clarify this point.*

3. The analysis correlating with m6A-mediated decay near the end of the manuscript is weak and should either be done more thoroughly or removed. To make a claim about m6A-mediated decay mechanisms, one should really be looking at the half-life of different transcripts, not just gene expression levels which will also be influenced by transcription and other processing steps.

*Reply: On the one hand, we acknowledge the reviewer’s point on the inherent weakness of drawing inferences with respect to stability based on steady state measurements. On the other hand, obtaining measurements of mRNA stability during meiosis is a major technical challenge that we have so far been unable to overcome, despite numerous attempts. Moreover, at least in the contexts of the analyses presented in yeast, the fact that a continuous trend is observed throughout the progression of meiosis, peaking at the same timepoint in which RNA methylation activity is at its maximum, presents - in our view - a compelling result. We should also emphasize that in this case the disadvantage of the more indirect readout (gene expression) is partially also an advantage: we go beyond demonstrating an impact only on mRNA stability (which has already been conducted in many prior studies), and instead show how the effects are manifested on gene expression levels, which is arguably a more interesting dimension. Based on the comment of this reviewer, we have decided, for now, to move the mammalian results (which lack a temporal resolution, and which is also more limited in terms of the ‘parental’ controls) to the Supplementary and to leave only the yeast results. However, we would also be happy to obtain editorial feedback on this point, and are open to the possibility of removing these results altogether, should this be the view of the editor.*

4. A more minor point that might be worth addressing is whether the fact that m6A methylation in yeast is restricted to meiosis may impact how conserved sites are across yeast. This is in contrast to the mammalian setting, where m6A methylation (particularly m6A-mediated mRNA decay) is a more broadly utilized regulatory mechanism across many different biological processes. That being said, this reviewer studies RNA modifications and is not an evolutionary biologist, so I would defer to experts in that area.

*Reply: This is an interesting hypothesis regarding the underpinnings of the different conservation patterns between the yeast hybrids (where many sites were subject to change) and mammalian sites (where much fewer sites changed). This hypothesis would also be consistent with the more limited evidence for selective constraints acting on m6A in yeast, in comparison to mammalian species 3. We have now added this thought to the discussion.*

*References*

*1. Liu, C. et al. Absolute quantification of single-base m6A methylation in the mammalian transcriptome using GLORI. Nat. Biotechnol. (2022) doi:10.1038/s41587-022-01487-9.*

*2. Xiao, Y.-L. et al. Transcriptome-wide profiling and quantification of N6-methyladenosine by enzyme-assisted adenosine deamination. Nat. Biotechnol. (2023) doi:10.1038/s41587-022-01587-6.*

*3. Liu, Z. & Zhang, J. Most m6A RNA Modifications in Protein-Coding Regions Are Evolutionarily Unconserved and Likely Nonfunctional. Mol. Biol. Evol. 35, 666–675 (2018).*

**Second round of review**

**Reviewer 1**

*The authors have addressed all of my questions. I am glad to recommend this paper for publication.*

**Reviewer 2**

*I appreciate the authors' thoughtful responses and clarifications related to the points made in my initial review. I am satisfied with their revisions and would be happy to have the manuscript published in its current form.*
